# Genomic studies of nitrogen-fixing rhizobial strains from *Phaseolus vulgaris* seeds and nodules

**DOI:** 10.1186/s12864-016-3053-z

**Published:** 2016-09-06

**Authors:** Humberto Peralta, Alejandro Aguilar, Rafael Díaz, Yolanda Mora, Gabriel Martínez-Batallar, Emmanuel Salazar, Carmen Vargas-Lagunas, Esperanza Martínez, Sergio Encarnación, Lourdes Girard, Jaime Mora

**Affiliations:** Centro de Ciencias Genómicas, Universidad Nacional Autónoma de México, Av. Universidad s/n, Chamilpa, Cuernavaca, Morelos CP 62210 Mexico

**Keywords:** Nitrogen fixation, Comparative genomics, Proteome

## Abstract

**Background:**

Rhizobia are soil bacteria that establish symbiotic relationships with legumes and fix nitrogen in root nodules. We recently reported that several nitrogen-fixing rhizobial strains, belonging to *Rhizobium phaseoli, R. trifolii, R. grahamii* and *Sinorhizobium americanum,* were able to colonize *Phaseolus vulgaris* (common bean) seeds. To gain further insight into the traits that support this ability, we analyzed the genomic sequences and proteomes of *R. phaseoli* (CCGM1) and *S. americanum* (CCGM7) strains from seeds and compared them with those of the closely related strains CIAT652 and CFNEI73, respectively, isolated only from nodules.

**Results:**

In a fine structural study of the *S. americanum* genomes, the chromosomes, megaplasmids and symbiotic plasmids were highly conserved and syntenic, with the exception of the smaller plasmid, which appeared unrelated. The symbiotic tract of CCGM7 appeared more disperse, possibly due to the action of transposases. The chromosomes of seed strains had less transposases and strain-specific genes. The seed strains CCGM1 and CCGM7 shared about half of their genomes with their closest strains (3353 and 3472 orthologs respectively), but a large fraction of the rest also had homology with other rhizobia. They contained 315 and 204 strain-specific genes, respectively, particularly abundant in the functions of transcription, motility, energy generation and cofactor biosynthesis. The proteomes of seed and nodule strains were obtained and showed a particular profile for each of the strains. About 82 % of the proteins in the comparisons appeared similar. Forty of the most abundant proteins in each strain were identified; these proteins in seed strains were involved in stress responses and coenzyme and cofactor biosynthesis and in the nodule strains mainly in central processes. Only 3 % of the abundant proteins had hypothetical functions.

**Conclusions:**

Functions that were enriched in the genomes and proteomes of seed strains possibly participate in the successful occupancy of the new niche. The genome of the strains had features possibly related to their presence in the seeds. This study helps to understand traits of rhizobia involved in seed adaptation.

**Electronic supplementary material:**

The online version of this article (doi:10.1186/s12864-016-3053-z) contains supplementary material, which is available to authorized users.

## Background

Rhizobia are saprophytic soil bacteria commonly studied for their ability to enter into nitrogen-fixing symbioses with legumes. The establishment of these symbioses by rhizobia, a collective term for strains from genera such as *Rhizobium*, *Sinorhizobium*, *Mesorhizobium* and *Bradyrhizobium*, involves the formation of organ-like structures on the legume roots (for recent reviews see references [1] and [2]). The rhizobia in the nodules are present in a metabolically differentiated form called bacteroids, which perform the reduction of atmospheric dinitrogen into ammonium. In exchange for dicarboxylic acids supplied from the plant, the bacteroids export the ammonium to the plant. Rhizobia have also been found inside legume non-nodular tissues such as roots, stems and pods [3–5]. There are also reports of endophytic rhizobia associated with *Arabidopsis*, wheat, maize, sugar cane, and rice [6–9]. Strains of endophytic *Rhizobium* were recently isolated from the tree species *Populus euphratica* and *P. deltoides* [10, 11].

Previously, we described several nitrogen-fixing rhizobial strains isolated from the interior of common bean seeds (*Phaseolus vulgaris*) [12]. We postulated that the vertical transmission of effective rhizobacteria in seeds expands the spectrum of their beneficial interactions with the host plants and has potential biotechnological application.

Given the increasing number of endophytic rhizobial isolates, it is worth determining which genetic traits are responsible for their ability to persist in plant tissues and discover if genomic differences exist among isolates able to persist in seeds. Despite the difficulties in assigning functions to novel genes, these analyses can measure changes in cellular physiology in response to genetic or environmental adaptations [13]. The model of our previous study was to compare closely related strains with different lifestyles by analyzing their genomes in addition to other approaches, with the aim of understanding how the strains have adapted to new niches. In a previous work, we reported an initial analysis of two genomes of seed-borne rhizobia corresponding to *Rhizobium phaseoli* (strain CCGM1) and *Sinorhizobium americanum* (strain CCGM7) species [12], but a thorough analysis was pending. The first species belongs to the symbiont most preferred by *P. vulgaris*, and the second to a recently described *Phaseolus* symbiont.

In this work we report the new genome sequence of an *S. americanum* strain, CFNEI73, isolated from nodules, and the improved sequence of strain CCGM7. We also compared the strains obtained from the interior of bean seeds, CCGM1 and CCGM7, with the strains from nodules, *R. phaseoli* CIAT652 [14] and *S. americanum* CFNEI73 [15], respectively. We analyzed their genomic sequences to infer the prevalence, identity and function of their orthologs, and also performed proteomic analyses to compare the abundance and function of proteins in seed-borne strains, compared with those from nodules*.*

## Methods

### Genome sequencing, assembly, and annotation of strain CFNEI73 and re-sequencing of CCGM7

DNA of strain CFNEI73 was extracted according to standard protocols and sequenced by Macrogen (Seoul, South Korea). A 3 kilobase pair (kb)-library was prepared and run on an Illumina HiSeq sequencer to obtain 100 base pair (bp)-mated pair reads. A total of 10,599,614 paired reads were obtained, and 4,629,584 remained after trimming. A second sequencing protocol was done with PacBio at the Duke Center for Genomic and Computational Biology (Durham, NC, USA) with a 10 kb-library, obtaining 731,017,143 reads, filtered to 612,800,193. Sequences obtained were mixed with the Illumina reads to enhance the accuracy of the final assembly with genome coverage of 71×. Assembly was performed with SMRT Analysis v2.3.0 (Pacific Biosciences) and SPAdes v3.5.0 [16]. Annotation was conducted with RAST v4.0 [17], with manual curation. Strain CCGM7 was re-sequenced with PacBio at the Duke Center for Genomic and Computational Biology, with a 10 kb-library, obtaining 1,147,065,864 reads, filtered to 998,800,19. Reads were mixed with those obtained previously with Illumina [12], with genome coverage of 121×. Assembly and annotation were done as for strain CFNEI73.

### Comparative genomic analysis

The comparison was performed in two ways: (i) by pairwise analysis in the case of strain-specific genes and (ii) by group analysis to calculate relatedness and sequence identity. The genome sequences were downloaded from GenBank with the following assembly accession numbers: *R. phaseoli* strains CIAT652 (GCA_000020265.1), CNPAF512 (GCA_000194195.2), and CCGM1 (GCA_000705615.1), *R. etli* CFN42 (GCA_000092045.1), *S. fredii* strains NGR234 (GCA_000018545.1), USDA257 (GCA_000265205.2) and HH103 (GCA_000283895.1). The most closely related species to *R. phaseoli* was *R. etli* and to *S. americanum* was *S. fredii*. Additional searches for rhizobial homologs were done with the nonredundant (nr) database. For the group comparison we used OrthoMCL version 2.0 [18] with default parameters, with BLAST (E value 1e^−5^; 30 % identity and 70 % overlap). Predicted ORFs with lengths <300 nt were discarded from the analysis of strain-specific genes. For the study of paralog families, the inparalog files were used. Synteny was determined with an in-house Perl program using the ortholog files from OrthoMCL as described previously [19]. Function was assigned using the extended annotation of clusters of orthologous groups (COG) tool [20]. Genomic average nucleotide identity (ANIm) was calculated with JSpecies [21]. Phylogeny of *nodD* was obtained with PhyML server (http://www.atgc-montpellier.fr/phyml/) using default parameters.

### Proteomic analysis

The strains were grown in liquid minimal medium (MM) containing succinate (10 mM) and ammonium chloride (10 mM) as carbon and nitrogen sources, respectively, for 8 h at 30 °C with 200 rpm shaking. The methods used for sample preparation, analytical and preparative two-dimensional (2D) polyacrylamide gel electrophoresis (PAGE), and image analysis were as described previously [22]. Briefly, pH gradients were determined by using a 2D SDS-PAGE standard (Sigma, United States). For the first dimension approximately 500 μg of total protein was loaded. The gels were stained with Coomassie blue R-250, and protein spots on the gels were detected at a resolution of 127 × 127 μm using a PDI image analysis system and PD-Quest software (Protein Databases, Inc., Huntington Station, NY). We were interested in spots that showed at least a 2-fold change with the corresponding protein in the other strain, and met the conditions of a statistical Student test (level of significance, 95 %). Fifty spots were selected per strain from Coomassie blue-stained preparative 2D gels, excised manually and prepared for mass spectrometry analysis [22]. Experiments were performed three times. Mass spectra were obtained using a Bruker Daltonics Autoflex (Bruker Daltonics, Billerica, MA) operated in the delayed extraction and reflectron mode. Spectra were externally calibrated using a peptide calibration standard (Bruker Daltonics 206095). Peak lists of the tryptic peptide masses were generated and searched against the NCBI nr databases or with Rhizobase (http://bacteria.kazusa.or.jp/rhizo/) using the Mascot search program (Matrix Science, Ltd., London United Kingdom). The isoelectric point and molecular weight of the proteins were calculated. Each of the proteins with spot concentration under the detection level was revised manually. A global proteome correlation between strains was calculated and expressed as percentage of similar proteins. Enrichment of gene ontology (GO) terms was done through the EVPedia server (http://student4.postech.ac.kr/evpedia2_xe/xe/) using the TopGO program v2.14.0 [23], with default parameters. Only the first five or 6 classes with the most significant *P* values, and exclusive terms for each strain, were included in the Table 4. The participation of the abundant proteins in metabolic pathways was graphed using the Biocyc site (http://biocyc.org/overviewsWeb/celOv.shtml#).

### Plasmid visualization by pulsed field gel electrophoresis (PFGE) and in Eckhardt gels

High-molecular-weight plasmids were visualized by PFGE, basically as described previously [24]. Gel electrophoresis was done in a Bio-Rad CHEF-DRIII system with the following conditions: one-sixth of the plug; initial switch time 800 s, final switch time 800 s, temperature 13.5 °C, field angle 106°, run time 64 h at 2.2 V cm^−1^. Plasmids were also visualized by the Eckhardt technique, as modified by Hynes and McGregor [25].

### Nucleotide accession numbers

The CFNEI73 genome has the following accession numbers at GenBank: CP013107 to CP013110 for chromosome, and plasmids a, b and c, respectively. For CCGM7, the sequences were registered under the accession numbers CP013051 to CP013054 for chromosome, and plasmids a, b and c, respectively.

## Results

The main objective of the work in the seed-borne rhizobial strains was to find specific differences that allow the bacteria to persist in legume seeds. The seed prevalence is very interesting due to the vertical transmission of the bacteria and its biotechnological potential, and represents a new paradigm in the *Rhizobium*-legume interaction. We consider that seed isolates form a distinctive new group of rhizobial strains that are adapted to endophytic life. Some strains still have the complete set of genes for nodulation and nitrogen fixation, but others lack some symbiotic genes (unpublished results). Apparently these strains are in an initial process of diversification and thus, must have phenotypic and genomic features that enable them to occupy the new niche.

The selection of strains for this study was based upon several factors. For example, the *R. phaseoli* CCGM1 seed strain belongs to the most common symbiont species of *P. vulgaris*, is a biotin auxotroph, has low pyruvate dehydrogenase (PDH) activity, shows decreased growth in subcultures of minimal medium, and high sensitivity in normal laboratory and storage conditions, but has normal symbiotic ability [12]. The *S. americanum* CCGM7 seed strain is a biotin prototroph, presents good stress resistance, high PDH activity, no growth dcrease in subcultures, and has high symbiotic performance [12]. Extraordinarily, this strain has genes new to symbiotic rhizobia such as *nifV* gene (encoding homocitrate synthase) that make it the first candidate for fixing nitrogen in free-living state. The nodule strains selected for comparison were the most closely related available. For *R. phaseoli*, the nodule strain CIAT652 was sequenced previously by us [26], and has been well characterized in our laboratory [14]. It is used as a biofertilizer for beans in Mexico and Central America. The *S. americanum* strain CFNEI73 was isolated in our Center from *Acacia* trees, and is also able to nodulate *P. vulgaris* and *Leucaena leucocephala* [15]. We sequenced this strain given that no *S. americanum* genome was available.

In the genomic studies, we firstly performed genomic comparisons of seed and nodule strains, looking for gene differences. A structural study was done to detect genome rearrangements. The presence of strain-specific genes and transposases was evaluated to explain genome rearrangements. Then, the genomic comparison helped to detect orthologs and strain-specific genes. A functional analysis on these genes was done to detect functions that possibly participate in the capability for seed persistance. The study of paralogs arose from the analysis of shared genes to detect signals of differential evolutionary trends. Finally, we obtained the proteomes of these strains, grown in minimal medium, to identify which proteins were most abundant in the seed strains, to deduce their function and participation in metabolism.

### Genome sequence of *S. americanum* CFNEI73

We obtained the genome sequence of *S. americanum* strain CFNEI73, characterized previously [15]. This strain is efficient for nodulation and nitrogen fixation with bean plants (data not shown). Strain CFNEI73 was reported to have three plasmids [15], but we observed only the two smallest by pulsed-field gel electrophoresis (PFGE) (Additional file 1: Figure S1). The CFNEI73 assembled genome consisted of a 3.7 Mb chromosome and three plasmids: pSamCFNEI73c (2177 Kb), pSamCFNEI73b or pSym (586 Kb), and pSamCFNEI73a (222 Kb), with 6466 predicted genes in total. Previously, we did not detect plasmids in the *S. americanum* strain CCGM7 [12]. Considering the CFNEI73 plasmid arrangement, we re-analyzed and re-sequenced strain CCGM7 and three plasmids were assembled: pSamCCGM7c (2249 Kb), pSamCCGM7b or pSym (547 Kb) and pSamCCGM7a (405 Kb) The two smallest were observed by PFGE (Additional file 1: Figure S1). The genomic features of strains CFNEI73 and CCGM7 are shown on the Table 1.

**Table 1:** Genomic features of *S. americanum* CCGM7 and CFNEI73 strains

Feature	CCGM7	CFNEI73
Genome size in bp (CDS^a^)	6,853,050 (6601)	6,751,508 (6466)
Size per replicon in bp (CDS):		
Plasmid a	405,481 (379)	222,756 (226)
Plasmid b (pSym)	547,106 (491)	586,526 (529)
Plasmid c	2,249,899 (2168)	2,177,502 (2045)
Chromosome	3,650,564 (3563)	3,764,724 (3666)
%G + C content (pa, pb, pc, chr)	60.0, 58.8, 62.1, 63.0	60.0, 59.0, 62.2, 63.0
% CDS with assigned function	77.3	76.3
Average CDS length, bp	887	898

^a^ *CDS* coding sequence

### Genomic comparison of seed-borne and nodule strains

#### Structural comparison of the genomes of S. americanum strains

**Fig. 1:**
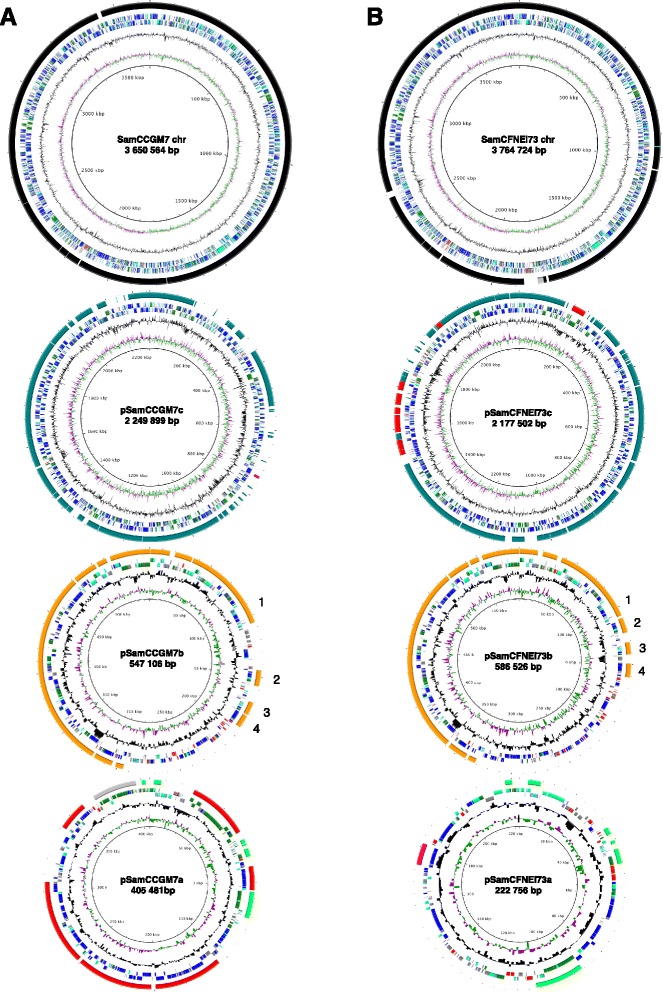
Schematic representation of the genomes of *S. americanum* strains CCGM7 and CFNEI73. **a** CCGM7. **b** CFNEI73. The circles represent, from *top* to *bottom*, the chromosome, plasmid c (megaplasmid), plasmid b (symbiotic plasmid) and plasmid a of each. From the innermost circle: GC skew, %GC content, ORF prediction with direction of transcription (color code according to the function: *dark blue* metabolism, *light blue* cellular processes*, green* information, *gray* unkown*, red* transposases and other mobile elements), structural comparison by synteny. Color code for synteny: *black*, synteny between chromosomes; *turquoise*, synteny between plasmids c; *orange*, synteny between plasmids b; *green*, syntenic segments between plasmids a. Other syntenic segments between replicons, as follow: CCGM7 pa with CFNEI73 chromosome *gray*; CCGM7 pc with CFNEI73 pa *pink*; and CCGM7 pa with CFNEI73 pc *red*

We obtained the ordered sequence of replicons of both *S. americanum* strains CCGM7 and CFNEI73 and performed a fine synteny analysis to detect rearrangements of genes with relative changed position. Figure 1 shows the ORF prediction for each replicon of the genomes and a synteny comparison. This analysis showed that the chromosomes were almost completely conserved between the strains and that the megaplasmids (plasmids c) and the symbiotic plasmids (plasmids b) showed extensive synteny, with 70–85 % total coverage. However, the smallest plasmid, pSamCFNEI73a, had only 18 % synteny with the CCGM7 genome, with the rest of the genes being strain-specific. The pSamCCGM7a was related to a segment of megaplasmid c of CFNEI73 (with 69 % synteny coverage). We also found that the cluster of genes for symbiosis on plasmid b were more dispersed in CCGM7, in comparison with CFNEI73, due to the insertion of strain-specific segments (see the positions of the numbers 1 to 4 in Fig. 1).

#### Abundance of transposases and strain-specific genes

The transposases are a key factor in the loss of synteny [19, 27, 28]. We found that the chromosomes of both seed-borne strains (CCGM1 and CCGM7) contained fewer transposases and integrases than the typical strains from nodules (Table 2). The plasmids contained from five to ten times more transposases, per megabase, than the chromosomes. As observed, the proportion of transposases per megabase remained almost constant in the plasmids, but was more reduced in the chromosomes of seed-strains. Since the incorporation of strain-specific genes could arise from recent events of transposition, we evaluated the number of these genes by pair comparisons (Table 2). The chromosomes of seed-borne strains had one-half to one-third less of strain-specific genes than the nodule strains, while the plasmids contained similar numbers. The pair of *Rhizobium* strains had twice the number of strain-specific genes in comparison to *Sinorhizobium*. Together, the results revealed a tendency to reduce both the number of transposases and the incorporation of strain-specific genes into the chromosomes of seed-borne strains.

**Table 2:** Transposases and strain-specific genes deduced by pairwise genome comparison in rhizobial strains

Transposases (Tn/Mb) ^a^	CCGM1	CIAT652	CCGM7	CFNEI73
Genome	67 (9.7)	86 (13.3)	85 (12.4)	96 (14.2)
By type of replicon:				
Chromosome	11 (2.5)	37 (8.2)	15 (4.1)	29 (7.7)
Plasmids	56 (23.4)	49 (25.3)	70 (21.9)	67 (22.4)
Homologs^b^	5402 (84.0)	5202 (84.6)	5783 (87.6)	5721 (88.5)
Strain-specific genes^c^	604 (9.4)	668 (10.9)	373 (5.7)	364 (5.6)
By type of replicon^d^:				
Chromosome	282 (6.6)	420 (9.7)	47 (1.3)	96 (2.6)
Plasmids	322 (14.7)	248 (14.5)	326 (10.7)	268 (9.6)

^a^Include integrases

^b^% of genome in parenthesis

^c^The rest of genes corresponded to short genes (<300 nt) which were discarded, by strain: CCGM1, 422; CIAT652, 279; CCGM7, 445; CFNEI73, 381

^d^% of replicon(s)

#### Ortholog detection and identity analysis

**Fig. 2:**
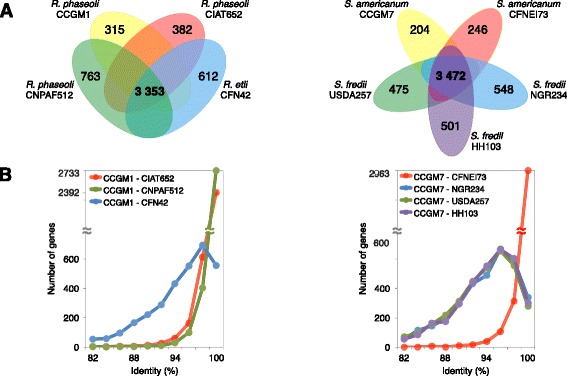
Gene content comparison between seed-borne rhizobial strains and nodule strains. **a** Venn diagram showing the number of gene clusters of shared orthologs and strain-specific genes among the genomes of *Rhizobium phaseoli-R. etli* and *Sinorhizobium americanum-S. fredii* strains. **b** Frequency distribution of identity percentages of shared orthologs, by group

A comparative analysis of the genomes of the isolates from bean seeds with related strains was performed. Strain CCGM1 was compared with *R. phaseoli* closest strains CIAT652 and CNPAF512 and then followed by strain CFN42 from the relative species *R. etli*. Strain CCGM7 was compared with *S. americanum* strain CFNEI73 and then with strains NGR234, HH103 and USDA257, from the closely related species *S. fredii*. Orthologs shared in each group were deduced. Figure 2a shows the number of orthologs shared among the strains and strain-specific genes. Only half of the genome of each organism had orthologs with the others; but many of the remaining genes also had homologs in several strains of Rhizobiales (not shown). In the tested groups, only 315 and 204 genes were found exclusively and without homologs in strains CCGM1 and CCGM7, respectively (Additional file 2: Table S1). Many of them had hypothetical function and others apparently are isozymes. The seed-strains showed lesser strain-specific genes than nodule strains.

To discern the relatedness of strains and the global identity of the shared orthologs, their sequency identities were calculated and a frequency distribution was obtained (Fig. 2b). The strain most closely related to CCGM1 was CNPAF512, with 98.4 % identity on average, followed by CIAT652 (97.9 %), and CFN42 (92.8 %). For CCGM7, the closest strain was CFNEI73 with average identity of 99.0 %, followed by the *S. fredii* strains HH103 (92.2 %), NGR234 (92.0 %) and USDA257 (91.8 %). We calculated the global average nucleotide identity (ANI) of the strains and the values obtained for these comparisons were in good concordance with the identity of shared orthologs (Additional file 3: Table S2) [29].

#### Function of orthologs and strain-specific genes

**Fig. 3:**
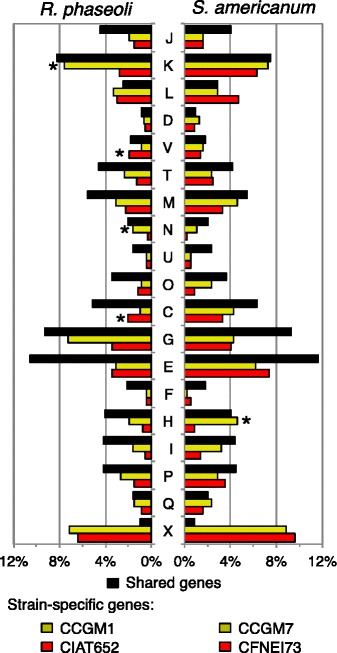
Functional classification of orthologs and strain-specific genes. *Left*, *Rhizobium phaseoli. Right, Sinorhizobium americanum. Yellow*, seed-borne strain. *Red*, nodule strain. *Black*, orthologs between the pair of strains. Distribution by COG functional categories. *Letters*: *J* Translation, ribosomal structure and biogenesis, *K* Transcription, *L* Replication, recombination and repair, *D* Cell cycle control, cell division, chromosome partitioning, *V* Defense mechanisms, *T* Signal transduction mechanisms, *M* Cell wall/membrane/envelope biogenesis, *N* Cell motility, *U* Intracellular trafficking, secretion, and vesicular transport, *O* Post-translational modification, protein turnover, and chaperones, *C* Energy production and conversion, *G* Carbohydrate transport and metabolism, *E* Amino acid transport and metabolism, *F* Nucleotide transport and metabolism, *H* Coenzyme transport and metabolism, *I* Lipid transport and metabolism, *P* Inorganic ion transport and metabolism, *Q* Secondary metabolites biosynthesis, transport, and catabolism, and *X* mobile elements. Genes with general function (*R*), poorly characterized (*S*) and not in COGs (−), were not included. *Asterisks* denote significant difference between seed-borne and nodule strains, with *p* > 0.05

A functional distribution was determined for orthologs and strain-specific genes of the seed-borne and nodule strains (Fig. 3). The proportions of shared orthologs of both comparisons had similar functional profile. The strain-specific genes of *R. phaseoli* CCGM1 were particularly abundant in transcription (COG class K) and cell motility (N) and for CIAT652 in defense (V) and energy generation (C). The strain-specific genes of CCGM7 were enriched in cofactor biosynthesis (H). These enriched functions possibly are important in the seed niche. Strain-specific genes of CFNEI73 appeared to have increased proportion only for replication and recombination (L).

#### Analysis of families of paralogs

**Fig. 4:**
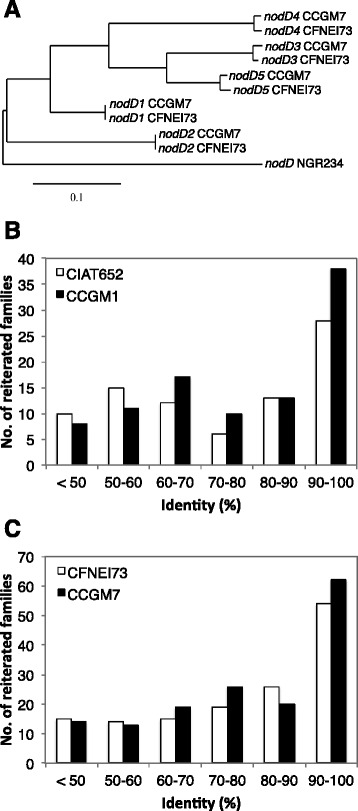
Phylogeny of *nodD* and identity of families of paralogs of the rhizobial strains. **a** Phylogenetic tree of *nodD* gene reiterations of *Sinorhizobium americanum* strains. *nodD* of *S. fredii* NGR234 was used as outgroup. **b** Frequency distribution of identity percentages, in classes of 10 %, among the members of families of paralogs in *R. phaseoli* strains CCGM1 and CIAT652. **c** Frequency distribution of identity percentages, in classes of 10 %, among the members of families of paralogs in *S. americanum* strains CCGM7 and CFNEI73

We found that CFNEI73 contained some genes that we initially described as unique in the CCGM7 genome [12]. For examples, the cluster of genes for hydrogenase (hydrogen uptake), the two RuBisCO genes (one of them associated to a complete cluster of genes of the Calvin cycle) and the *nifV* gene for the synthesis of homocitrate (the cofactor for nitrogenase, only found in free-living nitrogen fixers). Other unusual gene reiterations reported in strain CCGM7 were five *nodD* and three *nodA* reiterations, also present in the CFNEI73 genome. A phylogenetic tree showing the relatedness of the *nodD* reiterations is shown in Fig. 4a. Two paralogs appeared identical and the other three very similar. Given that, we extended the analysis to the genome content of both pairs of strains, looking for the families of paralogs and their identity level. In strain CCGM7 we found 156 groups of paralogs and 145 groups in CFNEI73. For CCGM1 and CIAT652, we found 101 groups and 86 groups, respectively. The identity among members of each group was calculated and a frequency distribution is shown in Fig. 4b and c. Despite a slight tendency to higher identity in the families of paralogs in the seed-borne strains, no significant statistical differences were found (ANOVA and Kruskal-Wallis H’s).

### Proteomes of the rhizobial seed isolates

We made proteome comparisons from cells grown in exponential phase (MM succinate-ammonium) to look for abundant specific proteins in the seed-borne strains in comparison with their close nodule relatives. CCGM1 and CIAT652 expressed about 725 and 710 proteins, or spots, respectively. By comparison, around 420 spots were the same in both strains; in contrast, 305 spots were found only in CCGM1 and 290 found only in CIAT652 (Additional file 4: Figure S2). The global correlation between the proteomes was 84 %. Table 3 lists 40 of the proteins with higher differential intensity identified in each strain. As can be observed, more proteins belonging to energy generation and translation appeared in CIAT652 strain; in contrast, more proteins for coenzyme and cofactor metabolism were abundant in CCGM1. Two hypothetical proteins were found in each strain and only five abundant proteins of strain CIAT652 had signals in CCGM1. We used the gene ontology (GO) enrichment terms tool to analyze the functional profiles of the abundant proteins in each of the strains. We found interesting differential profiles, as shown in the Table 4. For example, in CCGM1, enriched terms were cofactor and coenzyme binding and metabolism, transferases and oxidoreductases. For CIAT652, carbohydrate metabolism and sulfur compound metabolism with ATPase and pyrophosphatase, hydrolases and amino acyl-tRNA activities. The participation of abundant proteins in metabolic pathways was analyzed (Additional file 5: Figure S3A). As can be observed, pathways such as biosynthesis of carbohydrates, fatty acids and cofactors were better covered by the seed strain CCGM1, and the nodule strain CIAT652 had abundant proteins in the pathways of aminoacyl tRNA charging and carbohydrate degradation.

**Table 3:** Abundant proteins in the proteomes of seed-borne strains compared with nodule strains

Proteins of *R. phaseoli* CCGM1										
Spot no.	Gene ID	Protein	Function	COG	Mascot score	Sequence coverage	Matched peptides	Spot concentration^a^		Ratio
								CIAT652	CCGM1	
24	RLPCCGM1_c3018	SucB	Dihydrolipoamide succinyltransferase	C	115	27	13/28	-	679.8	-
41	RLPCCGM1_c0918	PdhA	Pyruvate dehydrogenase (acetyl-transferring) protein subunit alpha	C	71	25	9/36	503.7	1013.7	2.0
8	RLPCCGM1_p0657	RepA	Plasmid partitioning protein RepAb	D	73	28	10/32	21.8	77.3	3.5
5	RLPCCGM1_c0705	AldA	Alanine dehydrogenase	E	150	34	13/29	143.1	442.6	3.1
7	RLPCCGM1_c1335	SufS	Cysteine desulfurase	E	72	19	7/19	14.5	46.9	3.2
14	RLPCCGM1_p1587	FdhA	Glutathione-independent formaldehyde dehydrogenase	E	118	42	16/32	3.3	62.8	19.0
25	RLPCCGM1_c1870	IlvI	Acetolactate synthase 3 catalytic subunit	E	73	17	8/30	-	59.4	-
29	RLPCCGM1_c3812	ArgD	Acetylornithine transaminase	E	143	49	14/35	-	97.4	-
6	RLPCCGM1_c0701	CpdB	Bifunctional 2′,3′-cyclic nucleotide 2′-phosphodiesterase/3′-nucleotidase periplasmic protein	F	96	18	11/27	-	46.5	-
11	RLPCCGM1_c3249	PurH	Bifunctional phosphoribosylaminoimidazolecarboxamide formyltransferase/IMP cyclohydrolase	F	105	31	11/31	6.7	41.5	6.2
2	RLPCCGM1_c2616	Pgk	Phosphoglycerate kinase	G	118	36	11/25	17.5	41.3	2.4
9	RLPCCGM1_c2662	PykA	Pyruvate kinase	G	86	29	9/43	9.6	37.6	3.9
34	RLPCCGM1_c1984	-	Family 1 extracellular solute-binding protein	G	130	45	13/35	-	127.5	-
38	RLPCCGM1_c3763	FrcB	Fructose ABC transporter substrate-binding protein	G	75	25	5/8	-	156	-
40	RLPCCGM1_c2613	Gap	Glyceraldehyde 3-phosphate dehydrogenase	G	67	35	8/29	-	191.9	-
3	RLPCCGM1_c4187	Dxs	1-deoxy-D-xylulose-5-phosphate synthase	H	108	26	12/24	9.7	25.8	2.7
23	RLPCCGM1_c3273	CoaE	Dephospho-CoA kinase	H	57	23	5/23	-	53	-
26	RLPCCGM1_c4205	RibA	Riboflavin biosynthesis protein	H	69	24	7/21	-	75	-
36	RLPCCGM1_c3299	AhcY	S-adenosyl-L-homocysteine hydrolase	H	142	34	13/28	-	146.6	-
39	RLPCCGM1_c2569	SerC	Phosphoserine aminotransferase	H	113	43	12/39	-	190.4	-
17	RLPCCGM1_c3650	FadB	Enoyl-CoA hydratase	I	70	35	8/30	-	20.8	-
18	RLPCCGM1_c3059	-	Oxidoreductase	I	87	44	12/55	-	21	-
35	RLPCCGM1_c3416	FabB	3-oxoacyl-ACP synthase	I	89	33	12/49	-	127.6	-
1	RLPCCGM1_c1345	TyrS	Tyrosyl-tRNA synthetase	J	101	27	12/37	20.7	44	2.1
15	RLPCCGM1_c2760	-	Acetyltransferase	J	40	37	10/44	-	19.1	-
13	RLPCCGM1_c1935	MurD	UDP-N-acetylmuramoyl-L-alanyl-D-glutamate synthetase	M	139	31	13/26	-	15.2	-
33	RLPCCGM1_c2681	ExoN	UTP--glucose-1-phosphate uridylyltransferase	M	162	51	14/40	-	122.2	-
10	RLPCCGM1_c3962	FlaC	Flagellin C protein	N	80	32	8/29	29.6	178.8	6.0
21	RLPCCGM1_p1412	HtpG	Heat shock protein 90	O	103	45	13/35	-	31.3	-
22	RLPCCGM1_c1051	PpiA	Peptidyl-prolyl cis-trans isomerase A	O	55	30	4/29	-	38.1	-
31	RLPCCGM1_p1186	-	Glutathione S-transferase YghU	O	60	30	6/23	-	113.8	-
4	RLPCCGM1_p1923	KatG	Catalase	P	89	23	12/35	26.8	81.6	3.0
16	RLPCCGM1_c0709	SseA	Thiosulfate sulfurtransferase	P	138	55	12/34	-	20.7	-
27	RLPCCGM1_c2174	-	Methanol dehydrogenase regulator MoxR-like protein	R	108	52	14/48	-	76.8	-
28	RLPCCGM1_c3573	-	Oxidoreductase	R	80	37	12/48	-	76.8	-
30	RLPCCGM1_c2292	IdhA	Myo-inositol 2-dehydrogenase	R	87	44	12/55	-	102.8	-
19	RLPCCGM1_c4033	-	Isoprenylcysteine carboxyl methyltransferase	S	62	35	6/54	-	21.8	-
37	RLPCCGM1_c2285	-	Hypothetical protein	S	76	42	9/39	-	153.1	-
12	RLPCCGM1_p0713	-	Sensory box/GGDEF family protein	T	54	10	9/29	32.6	314.2	9.6
20	RLPCCGM1_p2036	CpaC	Pilus assembly protein	U	135	32	12/47	-	25.2	-
32	RLPCCGM1_p1459	-	Hypothetical protein	-	67	22	6/16	23	119.3	5.2
Proteins of *R. phaseoli* CIAT652										
Spot no.	Gene ID	Protein	Function	COG	Mascot score	Sequence coverage	Matched peptides	Spot concentration^a^		Ratio
								CIAT652	CCGM1	
47	RHECIAT_PC0000173	-	Putative oxidoreductase	C	132	21	15/27	6.5	-	-
62	RHECIAT_CH0002031	PdhA2	Pyruvate dehydrogenase subunit beta	C	114	37	18/39	51.5	-	-
67	RHECIAT_CH0004343	-	Aldehyde dehydrogenase	C	52	17	6/31	86.1	-	-
69	RHECIAT_CH0001680	NuoE1	NADH dehydrogenase subunit E	C	147	51	17/45	119.3	-	-
75	RHECIAT_CH0000039	PckA	Phosphoenolpyruvate carboxykinase	C	99	33	13/38	400.7	-	-
76	RHECIAT_CH0002032	PdhC	Dihydrolipoamide S-acetyltransferase	C	101	33	12/25	909.1	-	-
49	RHECIAT_PC0000423	-	Putative oligopeptide ABC transporter substrate-binding protein	E	56	8	7/23	7.6	2.9	2.6
50	RHECIAT_CH0003659	PotD	Spermidine/putrescine ABC transporter substrate-binding protein	E	60	25	7/24	243.5	23.9	10.2
60	RHECIAT_CH0002150	-	Peptide ABC transporter substrate-binding protein	E	218	44	22/38	39.9	-	-
72	RHECIAT_CH0001992	MetC	Cystathionine beta-lyase	E	81	38	9/22	190.4	-	-
73	RHECIAT_CH0000879	GuaB	Inosine 5′-monophosphate dehydrogenase	F	192	44	21/47	210.1	-	-
51	RHECIAT_CH0003979	LacZ2	Beta-D-galactosidase	G	129	18	11/14	16.8	-	-
55	RHECIAT_CH0000213	GpmA	Phosphoglyceromutase	G	214	84	18/52	23.7	-	-
61	RHECIAT_CH0003393	XylF	Xylose ABC transporter substrate-binding protein	G	113	32	10/41	46.7	-	-
64	RHECIAT_CH0003248	-	Omega amino acid--pyruvate transaminase	H	147	47	14/26	67.8	-	-
42	RHECIAT_CH0001955	AccC	Acetyl-CoA carboxylase biotin carboxylase subunit	I	77	33	9/26	795.2	364.4	2.2
53	RHECIAT_CH0003282	BdhA	D-beta-hydroxybutyrate dehydrogenase	I	81	28	6/8	19.3	-	-
48	RHECIAT_PA0000110	FusAa	Elongation factor G	J	55	16	8/27	6.8	-	-
54	RHECIAT_CH0004024	PrfA	Peptide chain release factor 1	J	119	41	14/40	19.9	-	-
58	RHECIAT_CH0001897	ValS	Valyl-tRNA synthetase	J	85	12	8/12	26.5	-	-
59	RHECIAT_CH0002285	ThrS	Threonyl-tRNA synthetase	J	124	23	16/34	31.3	-	-
65	RHECIAT_CH0000980	GlyS	Glycyl-tRNA synthetase subunit beta	J	192	38	23/37	71.6	-	-
66	RHECIAT_CH0002242	MetS	Methionyl-tRNA synthetase	J	105	26	10/22	72.1	-	-
57	RHECIAT_CH0001733	-	Nucleoside-diphosphate-sugar epimerase	M	57	19	5/22	26.4	-	-
74	RHECIAT_CH0003488	NoeJ	Mannose-1-phosphate guanylyltransferase (GDP) protein	M	145	48	18/52	243.4	-	-
46	RHECIAT_CH0001879	-	Peroxidase	O	67	35	8/29	119.2	23.7	5.0
52	RHECIAT_CH0001895	Pcm1	Protein-L-isoaspartate(D-aspartate) O-methyltransferase	O	93	38	6/16	19	-	-
63	RHECIAT_CH0002260	PpiD1	Peptidyl-prolyl cis-trans isomerase D signal peptide protein	O	57	16	6/14	67.7	-	-
70	RHECIAT_CH0004147	-	Nitrate/sulfonate/bicarbonate ABC transporter substrate-binding protein	P	106	42	11/29	127.6	-	-
71	RHECIAT_CH0002820	-	Ferrichrome ABC transporter substrate-binding protein	P	135	52	12/23	146.6	-	-
56	RHECIAT_PC0000681	VbsO	L-lysine 6-monooxygenase (NADPH) protein	Q	105	32	11/23	25.5	-	-
43	RHECIAT_CH0004212	-	ABC transporter substrate-binding protein	R	99	36	9/29	360.8	-	-
44	RHECIAT_CH0004405	-	Hypothetical protein	S	89	33	8/38	253.5	111.4	2.3
68	RHECIAT_CH0002473	-	Hypothetical protein	S	109	31	14/38	94.5	-	-
45	RHECIAT_CH0004199	TypA	GTP-binding protein TypA/BipA	T	151	34	21/42	215.3	-	-
Proteins of *S. americanum* CCGM7										
Spot no.	Gene ID	Protein	Function	COG	Mascot score	Sequence coverage	Matched peptides	Spot concentration^a^		Ratio
								CFNEI73	CCGM7	
2	SAMCCGM7_Ch3507	AcnA	Aconitate hydratase	C	69	17	11/31	-	185.6	-
3	SAMCCGM7_Ch3374	SucB	Dihydrolipoamide succinyltransferase	C	99	25	11/21	-	127	-
4	SAMCCGM7_Ch1973	FumC	Fumarate hydratase class II	C	100	21	10/20	-	105.8	-
8	SAMCCGM7_pC0039	TctC	Tricarboxylate transport protein	C	64	30	6/11	-	45.4	-
11	SAMCCGM7_Ch0047	PckA	Phosphoenolpyruvate carboxykinase	C	88	20	11/30	6.5	143.9	22.1
12	SAMCCGM7_Ch2624	Ald	Aldehyde dehydrogenase	C	65	17	7/18	-	17.3	-
20	SAMCCGM7_Ch3356	AtpD	ATP synthase subunit beta	C	111	38	13/34	41.4	407.9	9.9
28	SAMCCGM7_Ch1381	NuoG	NADH-quinone oxidoreductase subunit G	C	111	23	13/27	9.3	52.4	5.6
29	SAMCCGM7_Ch2523	AcoD	Acetaldehyde dehydrogenase 2	C	133	27	14/33	65.9	259.8	3.9
31	SAMCCGM7_Ch3358	AtpA	ATP synthase subunit alpha	C	97	29	14/41	78.5	290.4	3.7
16	SAMCCGM7_Ch1849	CarB	Carbamoyl-phosphate synthase large subunit	E	74	10	11/20	7.5	103.4	13.8
17	SAMCCGM7_Ch2552	LeuA	2-isopropylmalate synthase	E	118	27	13/23	-	11.4	-
18	SAMCCGM7_pA0276	-	Homoserine dehydrogenase	E	72	22	9/28	-	11.2	-
26	SAMCCGM7_Ch1267	GlyA	Pyridoxal-phosphate-dependent serine hydroxymethyltransferase	E	85	21	9/22	11.9	75.8	6.4
19	SAMCCGM7_Ch3100	-	5′-nucleotidase	F	122	22	12/19	11.1	120.3	10.8
35	SAMCCGM7_Ch3605	PurH	Bifunctional phosphoribosylaminoimidazolecarboxamide formyltransferase/IMP cyclohydrolase	F	80	23	10/31	140.6	278.9	2.0
24	SAMCCGM7_Ch3149	Pgm	Phosphoglucomutase Pgm	G	61	12	5/7	6.4	-	-
30	SAMCCGM7_Ch2630	ChvE	Multiple sugar-binding periplasmic receptor	G	55	24	6/25	34.2	127.5	3.7
32	SAMCCGM7_Ch3057	FbaB	Fructose-bisphosphate aldolase	G	70	32	9/34	27.2	95.8	3.5
33	SAMCCGM7_Ch3054	Gap	Glyceraldehyde-3-phosphate dehydrogenase	G	80	29	7/15	33.7	109.3	3.2
21	SAMCCGM7_Ch0041	AhcY	S-adenosyl-L-homocysteine hydrolase	H	81	26	10/29	21.2	169	8.0
14	SAMCCGM7_Ch1196	FabF	3-oxoacyl-ACP synthase	I	66	16	7/17	-	16.8	-
1	SAMCCGM7_Ch2527	RplY	50S ribosomal protein L25	J	199	72	17/32	-	498.9	-
7	SAMCCGM7_Ch1428	GatB	Aspartyl/glutamyl-tRNA(Asn/Gln) amidotransferase subunit B	J	72	18	7/19	-	59.6	-
23	SAMCCGM7_Ch0231	Pnp	Polyribonucleotide nucleotidyltransferase	J	122	29	17/38	35.5	270.8	7.6
9	SAMCCGM7_Ch0831	GroEL	Chaperonin	O	95	28	14/36	12.8	380.2	29.7
25	SAMCCGM7_pC0433	ClpV	Protease	O	63	15	10/32	-	7	-
5	SAMCCGM7_Ch0999	CysN	Sulfate adenylyltransferase subunit 1	P	121	45	15/44	-	65.2	-
13	SAMCCGM7_pC1570	-	Iron ABC transport system, solute-binding protein	P	89	19	17/11	-	17.1	-
22	SAMCCGM7_pC1145	-	Ferrichrome-iron receptor	P	149	19	13/16	-	7.8	-
27	SAMCCGM7_pC1571	-	Iron ABC transport system, solute-binding protein	P	105	26	11/23	20.6	123.5	6.0
34	SAMCCGM7_pC1766	Fct	Ferrichrysobactin receptor	P	174	35	18/31	2.6	7.4	2.8
6	SAMCCGM7_Ch2381	-	ABC transporter ATP-binding protein	R	126	31	17/30	-	60.4	-
10	SAMCCGM7_pA0100	VirB10	Type IV secretion system protein	U	150	32	12/19	-	23.8	-
15	SAMCCGM7_pC0430	ImpC	Type VI secretion system protein	U	96	23	10/21	14	206	14.7
Proteins of *S. americanum* CFNEI73										
Spot no.	Gene ID	Protein	Function	COG	Mascot score	Sequence coverage	Mached peptides	Spot concentration^a^		Ratio
								CFNEI73	CCGM7	
37	SAMCFNEI73_Ch3492	LpdA	Dihydrolipoamide dehydrogenase	C	134	37	13/23	48.2	-	-
44	SAMCFNEI73_Ch3194	-	Hypothetical protein	C	93	44	9/22	21.6	-	-
47	SAMCFNEI73_Ch0872	AtpF	ATP synthase subunit B’	C	94	35	7/18	51	3.4	15.0
53	SAMCFNEI73_Ch1106	-	Electron transfer flavoprotein-ubiquinone oxidoreductase	C	105	24	11/22	10.4	-	-
54	SAMCFNEI73_Ch0062	GlcB	Malate synthase G	C	156	27	14/19	15.1	1.7	8.9
57	SAMCFNEI73_Ch0755	MmsA	Methylmalonate-semialdehyde dehydrogenase	C	132	33	17/29	66.6	12.8	5.2
70	SAMCFNEI73_Ch1382	NuoD	NADH-quinone oxidoreductase subunit D	C	135	42	20/54	45.7	21	2.2
72	SAMCFNEI73_Ch3505	SdhA	Succinate dehydrogenase flavoprotein subunit	C	96	20	10/15	42.5	19.9	2.1
36	SAMCFNEI73_pC0436	FliY	Cystine-binding periplasmic protein	E	63	26	6/17	63.4	-	-
43	SAMCFNEI73_Ch1350	ArgC	N-acetyl-gamma-glutamyl-phosphate reductase	E	66	29	7/27	23.5	-	-
49	SAMCFNEI73_pC0934	HutU	Urocanate hydratase	E	93	21	13/34	13.2	-	-
51	SAMCFNEI73_Ch2492	IlvB	Acetolactate synthase large subunit	E	82	14	7/10	12.1	-	-
52	SAMCFNEI73_Ch0061	-	Hydantoinase/oxoprolinase family protein	E	71	17	7/16	10.5	-	-
59	SAMCFNEI73_Ch3399	MetH	Methionine synthase	E	208	25	24/29	4.6	-	-
63	SAMCFNEI73_Ch0442	DapD	2,3,4,5-tetrahydropyridine-2,6-carboxylate N-succinyltransferase	E	55	20	7/42	38.3	10.6	3.6
65	SAMCFNEI73_Ch2207	MetH2	Methionine synthase	E	79	28	8/37	25.4	7.9	3.2
69	SAMCFNEI73_Ch0208	-	Extracellular solute-binding protein, family 5	E	74	21	12/47	30	11	2.7
71	SAMCFNEI73_Ch1748	-	Peptide ABC transporter substrate-binding protein	E	93	24	13/34	32.5	15.1	2.2
62	SAMCFNEI73_Ch3182	Pgk	Phosphoglycerate kinase	G	113	41	12/26	146.7	39.3	3.7
66	SAMCFNEI73_Ch3273	GlgX	Glycogen debranching protein	G	131	23	12/18	6.7	2.1	3.2
58	SAMCFNEI73_pC1978	ThiC	Phosphomethylpyrimidine synthase	H	113	33	17/37	23.9	5	4.8
64	SAMCFNEI73_Ch1162	NadB	L-aspartate oxidase	H	156	31	15/23	33.7	9.7	3.5
48	SAMCFNEI73_pC0946	MmgC	Acyl-CoA dehydrogenase	I	83	32	13/40	13.9	-	-
46	SAMCFNEI73_Ch1475	FusA	Elongation factor G	J	93	22	12/24	15.8	-	-
42	SAMCFNEI73_Ch1028	-	ATPase component BioM of energizing module of biotin ECF transporter	L	136	40	15/36	23.8	-	-
39	SAMCFNEI73_pC1683	RkpQ	N-acylneuraminate-9-phosphate synthase	M	202	55	16/39	25.6	-	-
50	SAMCFNEI73_Ch1571	KdsA	2-dehydro-3-deoxyphosphooctonate aldolase	M	106	43	16/54	12.4	-	-
67	SAMCFNEI73_Ch2467	MurE	UDP-N-acetylmuramoyl-L-alanyl-D-glutamate--2,6-diaminopimelate ligase	M	166	34	13/19	26.3	8.7	3.0
40	SAMCFNEI73_Ch1644	Pcm	Protein-L-isoaspartate O-methyltransferase	O	140	57	11/20	24.9	-	-
61	SAMCFNEI73_Ch1368	LonA	Endopeptidase La	O	62	12	9/20	55	14.3	3.8
68	SAMCFNEI73_pC1783	-	Phosphate ABC transporter substrate-binding protein	P	78	27	7/24	104.4	36.3	2.9
45	SAMCFNEI73_Ch3296	-	Cobalamin synthesis protein/P47K family protein	R	69	25	9/37	17.9	-	-
55	SAMCFNEI73_Ch3670	-	Hypothetical protein	R	72	16	8/19	8.2	-	-
38	SAMCFNEI73_pB0529	TraI	Autoinducer synthesis protein	T	157	75	15/39	30.5	-	-
60	SAMCFNEI73_Ch1589	NtrC	Nitrogen assimilation regulatory protein	T	63	19	7/20	26.2	6	4.4
56	SAMCFNEI73_pB0525	TrbE	Conjugal transfer protein	U	177	27	20/32	7.9	-	-
41	SAMCFNEI73_pC1268	-	Peroxiredoxin	V	95	37	8/22	24.8	-	-

^a^In OD units. No value means the spot concentration was under the level of detection

For COG class definition, see legend of Fig. 3

**Table 4:** Gene ontology (GO) term enrichment for the abundant proteins of the proteomes of rhizobial strains

Strain GO ID	GO term	Annotated orthologous groups (OGs)	Annotated OGs in this list	Expected annotated OGs by random	*P*-value
CCGM1	Biological processes				
GO:0046395	Carboxylic acid catabolic process	425	12	0.4	3.60E-15
GO:0009063	Cellular amino acid catabolic process	221	10	0.21	8.50E-15
GO:0046365	Monosaccharide catabolic process	189	9	0.18	1.30E-13
GO:0051188	Cofactor biosynthetic process	624	12	0.59	3.30E-13
GO:0006732	Coenzyme metabolic process	710	12	0.67	1.50E-12
	Molecular function				
GO:0048037	Cofactor binding	993	12	0.88	3.50E-11
GO:0051287	NAD binding	69	5	0.06	4.40E-09
GO:0050662	Coenzyme binding	665	9	0.59	5.30E-09
GO:0016740	Transferase activity	7189	21	6.35	8.20E-08
GO:0016491	Oxidoreductase activity	3415	15	3.01	8.60E-08
CIAT652	Biological processes				
GO:0005996	Monosaccharide metabolic process	404	9	0.31	1.70E-11
GO:0046394	Carboxylic acid biosynthetic process	1074	11	0.83	2.70E-10
GO:0006790	Sulfur compound metabolic process	583	9	0.45	4.30E-10
GO:0044275	Cellular carbohydrate catabolic process	156	6	0.12	2.20E-09
GO:0009117	Nucleotide metabolic process	1446	11	1.12	5.90E-09
	Molecular function				
GO:0016787	Hydrolase activity	7866	21	5.7	3.00E-09
GO:0016462	Pyrophosphatase activity	1346	9	0.97	3.30E-07
GO:0042626	ATPase activity, coupled to transmembrane movement of substances	392	6	0.28	3.50E-07
GO:0016835	Carbon-oxygen lyase activity	411	6	0.3	4.60E-07
GO:0004022	Alcohol dehydrogenase (NAD) activity	23	3	0.02	6.10E-07
GO:0004812	Aminoacyl-tRNA ligase activity	106	4	0.08	1.10E-06
CCGM7	Biological processes				
GO:0046483	Heterocycle metabolic process	2459	17	1.72	3.50E-14
GO:0009117	Nucleotide metabolic process	1446	12	1.01	9.40E-11
GO:0015980	Energy derivation by oxidation of organic compounds	652	9	0.46	4.30E-10
GO:0010035	Response to inorganic substance	944	10	0.66	4.90E-10
GO:0006950	Response to stress	5245	18	3.67	5.80E-10
GO:0046686	Response to cadmium ion	271	7	0.19	6.60E-10
	Molecular function				
GO:0008266	Poly(U) RNA binding	22	4	0.01	1.10E-09
GO:0008187	Poly-pyrimidine tract binding	29	4	0.02	3.50E-09
GO:0046872	Metal ion binding	5534	16	3.63	4.80E-08
GO:0043169	Cation binding	5559	16	3.65	5.20E-08
GO:0003727	Single-stranded RNA binding	67	4	0.04	1.10E-07
GO:0017076	Purine nucleotide binding	1824	10	1.2	1.40E-07
CFNEI73	Biological processes				
GO:0044283	Small molecule biosynthetic process	1489	13	1.04	6.30E-12
GO:0016054	Organic acid catabolic process	425	9	0.3	9.70E-12
GO:0008652	Cellular amino acid biosynthetic process	491	9	0.34	3.50E-11
GO:0009081	Branched-chain amino acid metabolic process	55	5	0.04	4.00E-10
GO:0043648	Dicarboxylic acid metabolic process	148	6	0.1	8.30E-10
	Molecular function				
GO:0050662	Coenzyme binding	665	8	0.45	1.10E-08
GO:0050660	Flavin adenine dinucleotide binding	153	5	0.1	6.20E-08
GO:0016462	Pyrophosphatase activity	1346	9	0.91	1.80E-07
GO:0048037	Cofactor binding	993	8	0.67	2.40E-07
GO:0016817	Hydrolase activity, acting on acid anhydrides	1456	9	0.99	3.40E-07
GO:0051287	NAD binding	69	3	0.05	1.40E-05

Only exclusive terms for each strain are shown

The proteomes of strains CCGM7 and CFNEI73 showed 715 and 713 spots, respectively, sharing 491 spots. In this case, 224 were unique to CCGM7 and 222 unique to CFNEI73. The correlation between the proteomes was 82.6 %. Spots with differential abundance profiles were identified (Table 3). Only one protein with hypothetical function was found in CFNEI73. The GO analysis of the abundant proteins showed enriched terms in CCGM7 for stress response, energy generation and metal detoxification and polyU, pyrimidine and purine metabolisms and metal binding, and single strand RNA-binding (Table 4). For CFNEI73, enriched terms included branched amino acid and dicarboxylic acid metabolisms, with binding of coenzyme, flavin, cofactor and NAD as biological activities. The abundant proteins of the seed strain CCGM7 participated more in pathways of the biosynthesis of carbohydrates and fatty acids and glycolysis (Additional file 5: Figure S3B). The nodule strain CIAT652 had abundant proteins for amino acid biosynthesis, cell structure and carbohydrate degradation.

The analyzed strains showed in the proteomes about 700 proteins each, shared up to 490 with the related strain and from 222 to 305 were considered unique proteins. Based on the pair comparisons, about 50 of the most abundant proteins were chosen for each strain and analyzed. Only 5 proteins had hypothetical function. Even when the strains were growing at the same exponential rate, the proteins had different metabolic functions.

## Discussion

The seed-borne strains *R. phaseoli* CCGM1 and *S. americanum* CCGM7 described in this work were obtained through assays with noninoculated bean plants that nodulated and fixed nitrogen [12]. As described previously, the first non efficient strain tested was an *Agrobacterium tumefaciens* devoid of pTi and carrying instead a pSym derived from *R. etli* strain CFN42 [12]. Using these procedures, ten strains were isolated that showed plasmid profiles not observed previously. We reported that strain CCGM1 encoded several prophages (the firsts reported in *Rhizobium*), toxin/antitoxin pairs, queuosine, cellulosome anchoring system and other genes possibly related to the interaction with the plants [12]. The strain was a biotin auxotroph that showed a growth decline in serial subcultures, accumulated poly-beta-hydroxybutyrate (PHB) and had low pyruvate dehydrogenase (PDH) activity (as typical of some strains of its species), yet had optimal nodulation and nitrogen fixation ability [12].

Here, we compared the genomes and proteomes of rhizobial strains isolated only from nodules with isolates from bean seeds. The *R. phaseoli* strains were CIAT652 and CCGM1, respectively, and the *S. americanum* strains were CCGM7 and CFNEI73 [15]. This last strain was sequenced twice and, together with CCGM7 resequencing, allowed us to make a fine structural genome comparison (Fig. 1). The *S. americanum* strains each have three plasmids: a megaplasmid of about 2 Mb, the symbiotic plasmid ranging from 450 to 550 Kb, and a smaller plasmid between 200 and 400 kb. The main observation of the structural study was the high synteny of the chromosomes and the megaplasmids. However, their symbiotic plasmids showed important differences in the region surrounding the symbiotic gene clusters. Furthermore, the smallest plasmid of CCGM7 apparently derived from a segment of the megaplasmid, but the smallest plasmid of CFNEI73 was almost completely unrelated. The plasmids in these *S. americanum* strains were difficult to observe. However, it was easy to observe the plasmids of other strains of *S. americanum,* CCBAU051121 and CCBAU051127 [30]. In our previous report, we did not observe plasmids in strain CCGM7 [12]. However, given the genome assembly and the report that CFNEI73 contained three plasmids [15], additional efforts were made to detect its plasmids. To avoid the action of nucleases that possibly degrade the nucleic acids when the cells are lysed, the protocols were modified as described in Methods.

CFNEI73 also had some features that we previously found only in strain CCGM7: a *nifV* gene for homocitrate synthesis, hydrogenase uptake genes (*hup*) and two RubisCO clusters. The strains shared the three *nodA* and the five *nodD* reiterations (see the phylogeny in Fig. 4a), with some of them being identical and others having slight differences. The expansion of genetic families appears as an adaptative trait, as observed in *Leishmania* [31]. Also, we observed that chromosomes of seed-borne strains had less transposases and strain-specific genes in comparison to the typical strains, indicating reduced potential for rearrangement and possibly gene loss as a requisite for seed prevalence. This could be analogous to the genome reduction observed in obligate intracellular bacteria [32, 33].

The *S. americanum* strains had interesting metabolic abilities. CCGM7 had high PDH activity, grew without decline in serial cultures of minimal medium and, like CFNEI73, had the complete gene set for biotin synthesis, thus making them biotin prototrophs [12]. In the proteome analysis of abundant proteins, CCGM7 showed a protein set enriched for energy generation, response to stress, metal detoxification, translation and carbohydrate and ion metabolisms (Table 3). CFNEI73 proteins appeared enriched for amino acid transport and metabolism. The abundant proteins of seed strains participated in the metabolic pathways of biosynthesis of carbohydrates, fatty acids and cofactors. On the other hand, the nodule strains had better coverage of amino acyl tRNA charging, and biosyntheses of amino acids and cell structures (Additional file 5: Figure S3).

It is important to mention that only five hypothetical proteins were abundant in the proteins analyzed (from total 173). Apparently the main difference between the strains is related to the form in which the metabolism is performed, using the same main pathways. Also, 136 proteins had names, with specific function, and the rest 36 only generic functions. Only two proteins were abundant in both seed strains, namely PurH (Bifunctional phosphoribosyl amino imidazole carboxamide formyl transferase/IMP cyclo hydrolase) and SucB (Dihydrolipoamide succinyl transferase). This pair of proteins may be considered specific markers of the seed strains in minimal medium.

The differential metabolic functions of the identified abundant proteins were found even when the strains were growing at the same rate. Although the physiological meaning of these particular proteins in each strain can be matter of speculation, the data contribute to the characterization of the peculiarities of the strains. For example, the majority of abundant proteins (93 out 173) had no signal in the other strain, thus appearing as specific traits for each one. In *E. coli* it has been found that the core proteome is significantly enriched in nondiferentially expressed genes and depleted in differentially expressed genes [34].

The nodule strain CIAT652 had abundant proteins for energy generation, translation, and more dehydrogenases (related to redox and energy processes). We previously performed symbiotic and physiological characterization of this strain, qualifying it as a highly efficient strain [14, 26]. The abundant proteins of seed strain CCGM1 were enriched for synthesis of coenzymes and cofactors (Tables 3 and 4). The seed bacteria must develop great adaptative traits because the spermosphere is a new niche with high competition between seed borne and soil microorganisms occurring at the time of seed emergence [35].

It will be of interest to determine the host range of the *S. americanum* strains because they are relatively newly described species and their closest relatives belong to the very broad host range *S. fredii* strains NGR234 and USDA257, which can nodulate up to 112 and 79 legume species, respectively [36]. We have so far determined that CCGM7 can nodulate and fix nitrogen with *P. vulgaris* and *Medicago truncatula*; CFNEI73 can nodulate *Acacia farnesiana* [15] and *P. vulgaris*. As mentioned, the strains present five *nodD* reiterations and possibly these have a role in the host range. In a relevant recent paper, Del Cerro et al. determined that the five *nodD* genes of *R. tropici* CIAT899 were necessary to engage the microsymbiont in nodulation with different legume plants [37].

A factor that might be crucial for our ability to isolate rhizobia from seed was that the seeds were cropped from plants irrigated previously with nitrogen. Apparently, the seed strain is more adapted to the presence of nitrogen. On the other hand, nodulation is the main process by which *Rhizobium* colonizes the plants, but the seed niche is a less constrained environment that relaxes the selective pressure on the symbiotic genes. Although the strain persistence in seeds can represent an advantage for the plants given their potential metabolic capabilities, the seed rhizobia can also lose the symbiotic capability in the seed environment, without apparent consequences for the plant. Thus, originally the nodulation ability was a necessary feature for entry into the plants, but in the seed isolates it is not an essential feature.

## Conclusion

The seed-borne, nitrogen-fixing rhizobia strains represent an extended symbiotic model of the interaction with legume plants. Genomic differences such as rearrangement and reduction of transposases in the chromosomes possibly resulted from the adaptation to the seeds. Some functions such as stress response and biosynthesis of coenzymes, cofactors, carbohydrates and fatty acids appeared enriched in the seed strains. Comprehensive genomic studies, such as those presented here help to reveal global differences between the rhizobial seed strains and those isolated only from nodules.

## Additional files

Additional file 1: Figure S1. Plasmids of *S. americanum* strains visualized by pulsed field gel electrophoresis (PFGE). 1, CCGM7, 2 and 3, molecular weight marker (chromosomes of *Saccharomyces cerevisiae*, only some are denoted). 4, CFNEI73. Only the smallest plasmids were visible. (PDF 717 kb) Additional file 2: Table S1. Strain-specific genes of strains isolated from bean seeds, deduced by pairwise comparison with close relatives. Short genes (<300 nt) were discarded. (PDF 156 kb) Additional file 3: Table S2. Average nucleotide identity (ANIm) among the genomes of selected *Sinorhizobium* and *Rhizobium* strains. (PDF 34 kb) Additional file 4: Figure S2. Proteomes of *R. phaseoli* strains. A, CCGM1. B, CIAT652. Spots taken for analysis are encircled, in red, abundant proteins in the strain which were not visible in the other; in green, abundant proteins in the strain with counterpart in the other. Lines with arrows denote the direction of the 2D runs. (PDF 460 kb) Additional file 5: Figure S3. Metabolic reconstruction with BioCyc using the most abundant proteins of the strains detected in the proteome. A, *Rhizobium phaseoli* strains. Enzymes participating in metabolic pathways. Green boxes, the most abundant proteins from CCGM1 strain. Red boxes, the most abundant proteins from CIAT652 strain. B, *Sinorhizobium americanum* strains. Enzymes participating in metabolic pathways. Green boxes, the most abundant proteins from CCGM7 strain. Red boxes, the most abundant proteins from CFNEI73 strain. Brown were both coincided. The lists of proteins enable their use directly on the BioCyc page with the denoted organism. (−) denotes that no homolog was found in the strain. (PDF 355 kb)

## Abbreviations

ANIm: Genomic average nucleotide identity
Bp: Base pairs
COG: Cluster of orthologous groups
GO: Gene ontology
Kb: Kilo base pairs
NR: Nonredundant
PAGE: Polyacrylamide gel electrophoresis
PFGE: Pulsed-field gel electrophoresis
