# Supplementary material for: Genomic studies of nitrogen-fixing rhizobial strains from Phaseolus vulgaris seeds and nodules
Source: BMC Genomics. 2016 Sep 6;17(1):711. doi: 10.1186/s12864-016-3053-z (PMC5011921; doi:10.1186/s12864-016-3053-z)
Supplement: Additional file 1: Figure S1. — Plasmids of S. americanum strains visualized by pulsed field gel electrophoresis (PFGE). 1, CCGM7, 2 and 3, molecular weight marker (chromosomes of Saccharomyces cerevisiae, only some are denoted). 4, CFNEI73. Only the smallest plasmids were visible. (PDF 717 kb) [file 12864_2016_3053_MOESM1_ESM.pdf]

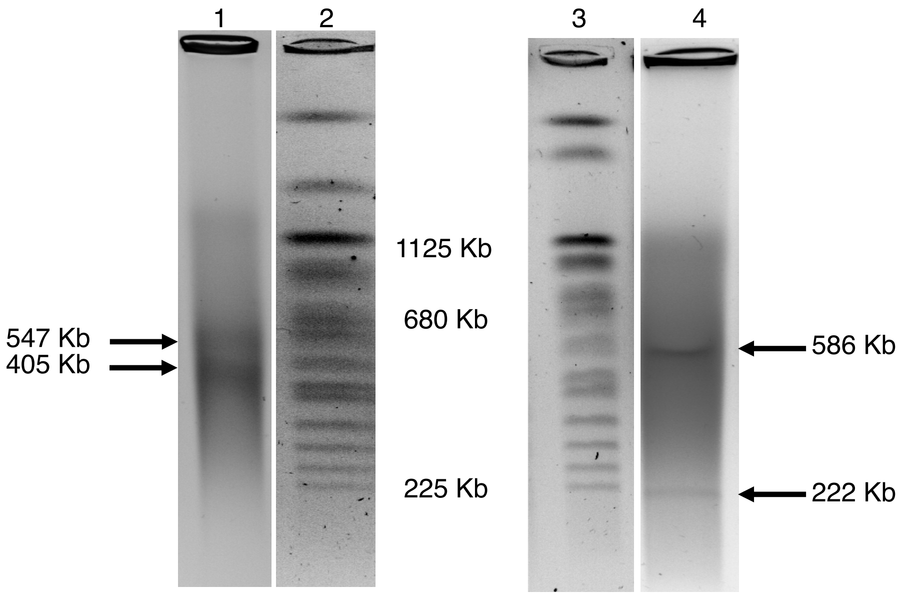

Supplementary Fig. 1. Plasmids of *S. americanum* strains visualized by pulsed field gel electrophoresis (PFGE). 1, CCGM7, 2 and 3, molecular weight marker (chromosomes of *Saccharomyces cerevisiae*, only some are denoted). 4, CFNEI73. Only the smallest plasmids were visible.
