# Supplementary material for: Genomic studies of nitrogen-fixing rhizobial strains from Phaseolus vulgaris seeds and nodules
Source: BMC Genomics. 2016 Sep 6;17(1):711. doi: 10.1186/s12864-016-3053-z (PMC5011921; doi:10.1186/s12864-016-3053-z)
Supplement: Additional file 2: Table S1. — Strain-specific genes of strains isolated from bean seeds, deduced by pairwise comparison with close relatives. Short genes (<300 nt) were discarded. (PDF 156 kb) [file 12864_2016_3053_MOESM2_ESM.pdf]

**Supplementary Table 1. Strain-specific genes of strains isolated from bean seeds, deduced by pairwise comparison with close relatives. Short genes (<300 nt) were discarded.**

***Rhizobium phaseoli* CCGM1**

| ID | Name | COG | Function |
|---|---|---|---|
| RLPCCGM1_c0006 | - | L | hypothetical protein |
| RLPCCGM1_c0032 | - | - | hypothetical protein |
| RLPCCGM1_c0035 | - | - | terminase small subunit protein |
| RLPCCGM1_c0036 | - | - | terminase large subunit protein |
| RLPCCGM1_c0037 | - | - | hypothetical protein |
| RLPCCGM1_c0038 | - | - | hypothetical protein |
| RLPCCGM1_c0039 | - | - | hypothetical protein |
| RLPCCGM1_c0040 | - | - | major virion structural protein |
| RLPCCGM1_c0041 | - | - | hypothetical protein |
| RLPCCGM1_c0042 | - | - | hypothetical protein |
| RLPCCGM1_c0043 | - | - | hypothetical protein |
| RLPCCGM1_c0044 | - | - | hypothetical protein |
| RLPCCGM1_c0045 | - | - | hypothetical protein |
| RLPCCGM1_c0046 | - | - | hypothetical protein |
| RLPCCGM1_c0047 | - | - | cellulosome anchoring protein |
| RLPCCGM1_c0048 | - | - | hypothetical protein |
| RLPCCGM1_c0049 | - | R | acetyltransferase |
| RLPCCGM1_c0050 | - | - | acyltransferase protein |
| RLPCCGM1_c0060 | - | K | LacI family transcriptional regulator |
| RLPCCGM1_c0065 | - | - | hypothetical protein |
| RLPCCGM1_c0071 | - | P | chloride channel protein |
| RLPCCGM1_c0072 | - | E | hypothetical protein |
| RLPCCGM1_c0121 | - | K | hypothetical protein |
| RLPCCGM1_c0122 | - | S | hypothetical protein |
| RLPCCGM1_c0132 | - | - | LysR family transcriptional regulator |
| RLPCCGM1_c0146 | - | - | hypothetical protein |
| RLPCCGM1_c0161 | - | - | hypothetical protein |
| RLPCCGM1_c0204 | - | S | hypothetical protein |
| RLPCCGM1_c0205 | - | S | hypothetical protein |
| RLPCCGM1_c0212 | - | I | short-chain dehydrogenase |
| RLPCCGM1_c0242 | - | E | membrane alanine aminopeptidase N |
| RLPCCGM1_c0244 | - | - | hypothetical protein |
| RLPCCGM1_c0286 | - | K | transcriptional regulator protein |
| RLPCCGM1_c0296 | - | - | porin |
| RLPCCGM1_c0321 | - | R | hypothetical protein |
| RLPCCGM1_c0397 | - | H | monooxygenase |
| RLPCCGM1_c0398 | - | - | PadR transcriptional regulator |
| RLPCCGM1_c0403 | - | L | regulatory inactivation of DnaA Hda protein |
| RLPCCGM1_c0407 | - | L | adenylate cyclase |
| RLPCCGM1_c0426 | *ribD* | H | 5-amino-6-(5-phosphoribosylamino)uracil reductase |
| RLPCCGM1_c0434 | - | - | hypothetical protein |
| RLPCCGM1_c0435 | - | - | hypothetical protein |
| RLPCCGM1_c0436 | - | - | hypothetical protein |
| RLPCCGM1_c0437 | - | - | acetyltransferase |
| RLPCCGM1_c0438 | - | Q | 3-demethylubiquinone-9 3-methyltransferase |
| RLPCCGM1_c0458 | - | M | cellulose synthase catalytic subunit |
| RLPCCGM1_c0459 | *celA* | M | cellulose synthase |
| RLPCCGM1_c0679 | - | - | periplasmic protein-like protein |
| RLPCCGM1_c0681 | - | E | ornithine cyclodeaminase |

| | | | |
|---|---|---|---|
| RLPCCGM1_c0682 | - | M | mandelate racemase/muconate lactonizing enzyme |
| RLPCCGM1_c0684 | - | L | DNA helicase |
| RLPCCGM1_c0685 | - | L | DNA helicase |
| RLPCCGM1_c0765 | - | - | hypothetical protein |
| RLPCCGM1_c0781 | - | L | transposase |
| RLPCCGM1_c0833 | - | - | hypothetical protein |
| RLPCCGM1_c0834 | - | - | hypothetical protein |
| RLPCCGM1_c0836 | - | - | hypothetical protein |
| RLPCCGM1_c0837 | - | - | hypothetical protein |
| RLPCCGM1_c0840 | - | - | phage protein |
| RLPCCGM1_c0841 | - | - | terminase large subunit protein |
| RLPCCGM1_c0842 | - | - | methyl-accepting chemotaxis protein |
| RLPCCGM1_c0843 | - | - | hypothetical protein |
| RLPCCGM1_c0844 | - | - | hypothetical protein |
| RLPCCGM1_c0845 | - | - | major virion structural protein |
| RLPCCGM1_c0846 | - | - | hypothetical protein |
| RLPCCGM1_c0847 | - | - | phage protein |
| RLPCCGM1_c0848 | - | - | hypothetical protein |
| RLPCCGM1_c0849 | - | - | hypothetical protein |
| RLPCCGM1_c0850 | - | - | hypothetical protein |
| RLPCCGM1_c0851 | - | - | hypothetical protein |
| RLPCCGM1_c0852 | - | - | flagellar protein FlgJ |
| RLPCCGM1_c0853 | - | - | hypothetical protein |
| RLPCCGM1_c0854 | - | - | hypothetical protein |
| RLPCCGM1_c0857 | - | - | hypothetical protein |
| RLPCCGM1_c0858 | - | - | phage antirepressor protein |
| RLPCCGM1_c0863 | - | - | hypothetical protein |
| RLPCCGM1_c0865 | - | - | succinoglycan biosynthesis protein exoI |
| RLPCCGM1_c0869 | - | L | phage integrase family protein |
| RLPCCGM1_c0927 | - | F | DNA topology modulation kinase FlaR-like protein |
| RLPCCGM1_c0939 | - | E | polar amino acid transport system permease protein |
| RLPCCGM1_c0940 | - | E | polar amino acid transport system permease protein |
| RLPCCGM1_c0958 | - | H | 2-octaprenyl-6-methoxyphenol hydroxylase |
| RLPCCGM1_c0959 | - | H | 2-octaprenyl-6-methoxyphenol hydroxylase |
| RLPCCGM1_c0964 | - | - | hypothetical protein |
| RLPCCGM1_c0978 | - | R | hypothetical protein |
| RLPCCGM1_c0980 | - | R | hypothetical protein |
| RLPCCGM1_c1074 | - | G | hypothetical protein |
| RLPCCGM1_c1131 | - | C | hypothetical protein |
| RLPCCGM1_c1132 | - | C | citrate synthase |
| RLPCCGM1_c1147 | - | R | hypothetical protein |
| RLPCCGM1_c1165 | - | L | transposase |
| RLPCCGM1_c1189 | - | S | hypothetical protein |
| RLPCCGM1_c1191 | - | - | hypothetical protein |
| RLPCCGM1_c1198 | - | T | two-component sensor histidine kinase |
| RLPCCGM1_c1206 | - | T | two-component response regulator protein |
| RLPCCGM1_c1208 | *cheB* | N | chemotaxis protein-glutamate methylesterase |
| RLPCCGM1_c1209 | *cheR* | N | chemotaxis methyltransferase |
| RLPCCGM1_c1219 | - | - | hypothetical protein |
| RLPCCGM1_c1236 | - | - | hypothetical protein |
| RLPCCGM1_c1238 | - | - | hypothetical protein |
| RLPCCGM1_c1242 | - | - | micrococcal nuclease (thermonuclease) |
| RLPCCGM1_c1244 | - | - | hypothetical protein |
| RLPCCGM1_c1246 | - | S | hypothetical protein |

| | | | |
|---|---|---|---|
| RLPCCGM1_c1247 | - | - | hypothetical protein |
| RLPCCGM1_c1248 | - | - | hypothetical protein |
| RLPCCGM1_c1249 | - | - | hypothetical protein |
| RLPCCGM1_c1250 | - | R | phage endolysin |
| RLPCCGM1_c1251 | - | - | hypothetical protein |
| RLPCCGM1_c1252 | - | - | hypothetical protein |
| RLPCCGM1_c1253 | - | - | hypothetical protein |
| RLPCCGM1_c1254 | - | - | hypothetical protein |
| RLPCCGM1_c1255 | - | - | hypothetical protein |
| RLPCCGM1_c1257 | - | - | hypothetical protein |
| RLPCCGM1_c1258 | - | - | hypothetical protein |
| RLPCCGM1_c1259 | - | - | hypothetical protein |
| RLPCCGM1_c1260 | - | - | hypothetical protein |
| RLPCCGM1_c1262 | - | - | hypothetical protein |
| RLPCCGM1_c1263 | - | - | hypothetical protein |
| RLPCCGM1_c1264 | - | - | hypothetical protein |
| RLPCCGM1_c1265 | - | - | hypothetical protein |
| RLPCCGM1_c1266 | - | - | hypothetical protein |
| RLPCCGM1_c1268 | - | - | SAM-dependent methyltransferase |
| RLPCCGM1_c1269 | - | - | hypothetical protein |
| RLPCCGM1_c1270 | - | - | hypothetical protein |
| RLPCCGM1_c1271 | - | - | hypothetical protein |
| RLPCCGM1_c1272 | - | - | phage protein |
| RLPCCGM1_c1273 | - | - | phage protein |
| RLPCCGM1_c1274 | - | - | hypothetical protein |
| RLPCCGM1_c1275 | - | O | peptidase S49 |
| RLPCCGM1_c1276 | - | R | phage portal protein, lambda family |
| RLPCCGM1_c1278 | - | - | hypothetical protein |
| RLPCCGM1_c1279 | - | R | bacteriophage tail assembly protein |
| RLPCCGM1_c1280 | - | - | hypothetical protein |
| RLPCCGM1_c1281 | - | K | hypothetical protein |
| RLPCCGM1_c1282 | - | - | hypothetical protein |
| RLPCCGM1_c1283 | - | - | hypothetical protein |
| RLPCCGM1_c1289 | - | - | hypothetical protein |
| RLPCCGM1_c1290 | - | - | hypothetical protein |
| RLPCCGM1_c1292 | - | - | hypothetical protein |
| RLPCCGM1_c1294 | - | - | hypothetical protein |
| RLPCCGM1_c1296 | - | K | phage repressor |
| RLPCCGM1_c1299 | - | - | hypothetical protein |
| RLPCCGM1_c1302 | - | - | hypothetical protein |
| RLPCCGM1_c1303 | - | - | hypothetical protein |
| RLPCCGM1_c1315 | *elaA* | R | protein ElaA |
| RLPCCGM1_c1316 | - | S | hypothetical protein |
| RLPCCGM1_c1388 | - | S | hypothetical protein |
| RLPCCGM1_c1389 | - | S | hypothetical protein |
| RLPCCGM1_c1402 | - | S | hypothetical protein |
| RLPCCGM1_c1417 | - | - | tRNA synthetase class II (G H P and S) |
| RLPCCGM1_c1419 | - | T | sensory box/GGDEF family protein |
| RLPCCGM1_c1424 | - | M | muconate cycloisomerase |
| RLPCCGM1_c1430 | - | L | hypothetical protein |
| RLPCCGM1_c1557 | *cobC* | E | cobalamin biosynthesis protein, pyridoxal-phosphate-dependent aminotransferase |
| RLPCCGM1_c1576 | - | - | LysR family transcriptional regulatory protein |
| RLPCCGM1_c1585 | - | - | hypothetical protein |

| | | | |
|---|---|---|---|
| RLPCCGM1_c1601 | - | G | dihydroxyacetone kinase, ATP-dependent |
| RLPCCGM1_c1602 | - | S | hypothetical protein |
| RLPCCGM1_c1607 | - | R | methyltransferase |
| RLPCCGM1_c1608 | - | P | iron-chelator utilization protein |
| RLPCCGM1_c1614 | - | H | oxidoreductase |
| RLPCCGM1_c1615 | - | - | transcriptional regulator, TetR family |
| RLPCCGM1_c1623 | - | - | hypothetical protein |
| RLPCCGM1_c1646 | - | - | hypothetical protein |
| RLPCCGM1_c1658 | - | L | transposase |
| RLPCCGM1_c1712 | - | - | hypothetical protein |
| RLPCCGM1_c1731 | - | - | hypothetical protein |
| RLPCCGM1_c1732 | - | - | hypothetical protein |
| RLPCCGM1_c1741 | - | - | hypothetical protein |
| RLPCCGM1_c1750 | - | K | GntR family transcriptional regulator |
| RLPCCGM1_c1797 | - | - | hypothetical protein |
| RLPCCGM1_c1809 | - | S | hypothetical protein |
| RLPCCGM1_c1821 | - | - | hypothetical protein |
| RLPCCGM1_c1823 | - | K | transcriptional regulator, TetR family |
| RLPCCGM1_c1837 | - | J | hypothetical protein |
| RLPCCGM1_c1838 | - | J | hypothetical protein |
| RLPCCGM1_c1839 | - | J | tetracycline resistance protein |
| RLPCCGM1_c1864 | - | - | hypothetical protein |
| RLPCCGM1_c1874 | - | K | GntR family transcriptional regulator |
| RLPCCGM1_c1922 | - | - | hypothetical protein |
| RLPCCGM1_c1991 | - | - | hypothetical protein |
| RLPCCGM1_c2011 | - | - | hypothetical protein |
| RLPCCGM1_c2012 | - | L | adenine specific DNA methyltransferase, D12 class |
| RLPCCGM1_c2014 | - | - | hypothetical protein |
| RLPCCGM1_c2015 | - | - | hypothetical protein |
| RLPCCGM1_c2016 | - | S | tail protein |
| RLPCCGM1_c2017 | - | S | phage FluMu protein gp47 |
| RLPCCGM1_c2018 | - | - | hypothetical protein |
| RLPCCGM1_c2019 | - | - | Mu-like prophage protein gp45-like protein |
| RLPCCGM1_c2020 | - | R | Mu P family protein |
| RLPCCGM1_c2021 | - | - | Mu-like prophage DNA circulation protein |
| RLPCCGM1_c2022 | - | - | hypothetical protein |
| RLPCCGM1_c2024 | - | - | hypothetical protein |
| RLPCCGM1_c2025 | - | - | hypothetical protein |
| RLPCCGM1_c2026 | - | R | Mu tail sheath family protein |
| RLPCCGM1_c2027 | - | - | hypothetical protein |
| RLPCCGM1_c2028 | - | - | hypothetical protein |
| RLPCCGM1_c2029 | - | S | hypothetical protein |
| RLPCCGM1_c2030 | - | - | hypothetical protein |
| RLPCCGM1_c2031 | - | - | hypothetical protein |
| RLPCCGM1_c2032 | - | R | Mu-like prophage FluMu major head subunit |
| RLPCCGM1_c2033 | - | - | hypothetical protein |
| RLPCCGM1_c2034 | - | R | Mu-like prophage I protein-like protein |
| RLPCCGM1_c2035 | - | R | phage virion morphogenesis protein |
| RLPCCGM1_c2036 | - | S | phage (Mu-like) virion morphogenesis protein |
| RLPCCGM1_c2037 | - | S | Mu-like prophage FluMu protein gp29 |
| RLPCCGM1_c2038 | - | R | Mu-like prophage FluMu protein gp28 |
| RLPCCGM1_c2039 | - | - | Mu-like prophage FluMu protein gp27 |
| RLPCCGM1_c2040 | - | - | phage protein |
| RLPCCGM1_c2041 | - | - | hypothetical protein |

| | | | |
|---|---|---|---|
| RLPCCGM1_c2042 | - | - | hypothetical protein |
| RLPCCGM1_c2044 | - | - | hypothetical protein |
| RLPCCGM1_c2045 | - | - | hypothetical protein |
| RLPCCGM1_c2046 | - | S | protein gp16 |
| RLPCCGM1_c2047 | - | - | hypothetical protein |
| RLPCCGM1_c2049 | - | - | hypothetical protein |
| RLPCCGM1_c2053 | - | R | bacteriophage DNA transposition protein B |
| RLPCCGM1_c2054 | - | L | transposase |
| RLPCCGM1_c2055 | - | - | hypothetical protein |
| RLPCCGM1_c2056 | *parB* | K | chromosome partitioning protein parB |
| RLPCCGM1_c2057 | - | - | hypothetical protein |
| RLPCCGM1_c2058 | - | - | hypothetical protein |
| RLPCCGM1_c2060 | - | - | hypothetical protein |
| RLPCCGM1_c2061 | - | - | hypothetical protein |
| RLPCCGM1_c2062 | - | - | hypothetical protein |
| RLPCCGM1_c2090 | - | - | hypothetical protein |
| RLPCCGM1_c2198 | - | S | extensin-like protein |
| RLPCCGM1_c2300 | - | P | potassium channel protein |
| RLPCCGM1_c2329 | - | - | transmembrane protein |
| RLPCCGM1_c2332 | *pssR* | R | acetyltransferase |
| RLPCCGM1_c2333 | *pssM* | - | ketal pyruvate transferase |
| RLPCCGM1_c2335 | *pssK* | G | exopolysaccharide polymerization protein |
| RLPCCGM1_c2336 | *pssJ* | - | galactosyltransferase |
| RLPCCGM1_c2363 | - | M | mannose-1-phosphate guanylyltransferase |
| RLPCCGM1_c2364 | *noeJ* | G | mannose-1-phosphate guanylyltransferase (GDP) protein |
| RLPCCGM1_c2410 | - | - | hypothetical protein |
| RLPCCGM1_c2536 | - | E | spermidine Putrescine ABC transporter permease component potC |
| RLPCCGM1_c2609 | - | S | hypothetical protein |
| RLPCCGM1_c2657 | - | K | transcriptional regulator IrhA |
| RLPCCGM1_c2751 | - | K | acetyltransferase |
| RLPCCGM1_c3178 | - | E | extracellular solute-binding protein |
| RLPCCGM1_c3185 | - | J | amino acid--(acyl-carrier-protein) ligase |
| RLPCCGM1_c3186 | - | - | serine--tRNA ligase protein |
| RLPCCGM1_c3201 | - | G | transport protein |
| RLPCCGM1_c3278 | *mltA* | M | membrane-bound lytic murein transglycosylase |
| RLPCCGM1_c3335 | - | L | Superfamily I DNA/RNA helicase protein |
| RLPCCGM1_c3336 | - | L | Superfamily I DNA/RNA helicase protein |
| RLPCCGM1_c3373 | - | - | hypothetical protein |
| RLPCCGM1_c3374 | - | L | integrase |
| RLPCCGM1_c3377 | - | - | integrase family protein |
| RLPCCGM1_c3379 | - | - | signal-transduction protein with CBS domain protein |
| RLPCCGM1_c3397 | - | - | hypothetical protein |
| RLPCCGM1_c3424 | *infB* | J | translation initiation factor IF-2 |
| RLPCCGM1_c3462 | *ftsK* | D | cell division protein |
| RLPCCGM1_c3595 | - | E | hypothetical protein |
| RLPCCGM1_c3726 | - | I | fatty acid desaturase |
| RLPCCGM1_c3727 | - | K | ArsR family transcriptional regulator |
| RLPCCGM1_c3739 | - | - | hypothetical protein |
| RLPCCGM1_c3750 | - | K | TfoX domain protein |
| RLPCCGM1_c3753 | - | - | two-component response regulator protein |
| RLPCCGM1_c3754 | - | S | two-component response regulator protein |
| RLPCCGM1_c3849 | *cspA* | K | cold shock protein |
| RLPCCGM1_c3880 | - | - | hypothetical protein |
| RLPCCGM1_c4039 | - | - | methyltransferase type 11 |

| | | | |
|---|---|---|---|
| RLPCCGM1_c4052 | - | M | glycosyl transferase |
| RLPCCGM1_c4053 | - | Q | methyltransferase type 11 |
| RLPCCGM1_c4055 | - | - | hypothetical protein |
| RLPCCGM1_c4056 | - | - | sugar transferase |
| RLPCCGM1_c4057 | - | M | glycosyl transferase |
| RLPCCGM1_c4100 | - | O | hypothetical protein |
| RLPCCGM1_c4131 | - | D | scaffold protein for (4Fe-4S) cluster assembly ApbC, MRP-like protein |
| RLPCCGM1_c4178 | - | I | acyl-CoA dehydrogenase |
| RLPCCGM1_c4179 | - | I | cyl-CoA dehydrogenase protein |
| RLPCCGM1_c4207 | - | T | diguanylate cyclase/phosphodiesterase with PAS/PAC sensor(s) |
| RLPCCGM1_c4228 | - | F | hypothetical protein |
| RLPCCGM1_c4264 | - | D | hypothetical protein |
| RLPCCGM1_c4269 | - | - | hypothetical protein |
| RLPCCGM1_c4273 | - | - | hypothetical protein |
| RLPCCGM1_c4277 | - | - | acetyltransferase |
| RLPCCGM1_c4278 | - | S | transcriptional regulator |
| RLPCCGM1_c4288 | - | - | transmembrane protein |
| RLPCCGM1_c4303 | - | Q | hypothetical protein |
| RLPCCGM1_p0013 | - | R | phenazine biosynthesis protein PhzF |
| RLPCCGM1_p0014 | - | P | manganese transport protein |
| RLPCCGM1_p0020 | *cheR* | T | chemotaxis protein methyltransferase CheR |
| RLPCCGM1_p0022 | - | T | hypothetical protein |
| RLPCCGM1_p0023 | - | T | sensor histidine kinase |
| RLPCCGM1_p0026 | - | - | transcriptional regulator, PadR family |
| RLPCCGM1_p0027 | - | C | zinc-binding dehydrogenase |
| RLPCCGM1_p0028 | - | - | hypothetical protein |
| RLPCCGM1_p0033 | - | - | hypothetical protein |
| RLPCCGM1_p0036 | - | - | hypothetical protein |
| RLPCCGM1_p0037 | - | - | hypothetical protein |
| RLPCCGM1_p0038 | - | S | hicB family protein |
| RLPCCGM1_p0040 | - | M | potassium efflux system KefA protein |
| RLPCCGM1_p0041 | - | P | arylsulfatase |
| RLPCCGM1_p0044 | - | - | hypothetical protein |
| RLPCCGM1_p0048 | - | - | hypothetical protein |
| RLPCCGM1_p0049 | - | - | hypothetical protein |
| RLPCCGM1_p0050 | - | - | hypothetical protein |
| RLPCCGM1_p0052 | - | - | phosphoglycerate kinase |
| RLPCCGM1_p0054 | - | - | cysteine rich repeat domain protein |
| RLPCCGM1_p0055 | - | - | OmpA/MotB domain protein |
| RLPCCGM1_p0056 | - | - | hypothetical protein |
| RLPCCGM1_p0057 | - | P | hypothetical protein |
| RLPCCGM1_p0060 | - | S | hypothetical protein |
| RLPCCGM1_p0067 | - | - | hypothetical protein |
| RLPCCGM1_p0085 | - | T | adenylate/guanylate cyclase |
| RLPCCGM1_p0087 | - | - | hypothetical protein |
| RLPCCGM1_p0088 | - | S | hypothetical protein |
| RLPCCGM1_p0090 | - | - | hypothetical protein |
| RLPCCGM1_p0094 | - | I | hypothetical protein |
| RLPCCGM1_p0105 | - | E | prolyl endopeptidase |
| RLPCCGM1_p0108 | - | - | transposase |
| RLPCCGM1_p0117 | - | S | gifsy-2 prophage protein |
| RLPCCGM1_p0119 | - | S | gifsy-2 prophage protein |
| RLPCCGM1_p0122 | - | L | integrase/recombinase clustered with segregation and condensation protein B |

| | | | |
|---|---|---|---|
| RLPCCGM1_p0123 | - | L | integrase/recombinase clustered with segregation and condensation protein B |
| RLPCCGM1_p0125 | - | S | hypothetical protein |
| RLPCCGM1_p0134 | - | G | TRAP-type C4-dicarboxylate transport system, periplasmic component |
| RLPCCGM1_p0135 | - | - | hypothetical protein |
| RLPCCGM1_p0145 | - | R | hydrolase of the alpha/beta superfamily |
| RLPCCGM1_p0151 | - | E | FAD dependent oxidoreductase |
| RLPCCGM1_p0153 | - | E | saccharopine dehydrogenase |
| RLPCCGM1_p0154 | - | M | hypothetical protein |
| RLPCCGM1_p0156 | - | F | hypothetical protein |
| RLPCCGM1_p0157 | - | - | hypothetical protein |
| RLPCCGM1_p0158 | - | K | TetR family transcriptional regulator |
| RLPCCGM1_p0161 | *kce* | S | 3-keto-5-aminohexanoate cleavage enzyme |
| RLPCCGM1_p0162 | - | - | hypothetical protein |
| RLPCCGM1_p0167 | - | S | hypothetical protein |
| RLPCCGM1_p0168 | - | - | hypothetical protein |
| RLPCCGM1_p0181 | - | - | TetR family transcriptional regulator |
| RLPCCGM1_p0183 | - | I | oxidoreductase |
| RLPCCGM1_p0246 | - | E | ABC transporter, periplasmic substrate-binding protein |
| RLPCCGM1_p0247 | - | E | ABC transporter, periplasmic substrate-binding protein |
| RLPCCGM1_p0282 | - | - | transcriptional regulator protein |
| RLPCCGM1_p0287 | - | Q | isochorismatase hydrolase |
| RLPCCGM1_p0302 | - | G | inner membrane component of tripartite multidrug resistance system |
| RLPCCGM1_p0318 | - | R | NADP oxidoreductase coenzyme F420-dependent |
| RLPCCGM1_p0343 | - | S | hypothetical protein |
| RLPCCGM1_p0344 | - | - | hypothetical protein |
| RLPCCGM1_p0378 | - | - | hypothetical protein |
| RLPCCGM1_p0439 | - | R | hydrolase |
| RLPCCGM1_p0443 | *hutU* | E | urocanate hydratase |
| RLPCCGM1_p0461 | - | Q | ubiquinone/menaquinone biosynthesis methyltransferase ubiE |
| RLPCCGM1_p0470 | - | S | hypothetical protein |
| RLPCCGM1_p0471 | - | - | hypothetical protein |
| RLPCCGM1_p0472 | - | - | hypothetical protein |
| RLPCCGM1_p0497 | - | - | hypothetical protein |
| RLPCCGM1_p0498 | - | R | ankyrin repeat-rich membrane spanning protein |
| RLPCCGM1_p0503 | - | E | oligopeptide ABC transporter ATP-binding protein |
| RLPCCGM1_p0520 | - | T | multi-sensor hybrid histidine kinase |
| RLPCCGM1_p0526 | *copD* | P | copper resistance protein D |
| RLPCCGM1_p0527 | - | R | hypothetical protein |
| RLPCCGM1_p0529 | - | S | hypothetical protein |
| RLPCCGM1_p0531 | - | L | hypothetical protein |
| RLPCCGM1_p0538 | - | L | hypothetical protein |
| RLPCCGM1_p0539 | - | L | transposase |
| RLPCCGM1_p0542 | - | L | transposase |
| RLPCCGM1_p0543 | - | L | insertion sequence IS21 ATP-binding protein |
| RLPCCGM1_p0549 | - | L | insertion sequence ATP-binding protein |
| RLPCCGM1_p0551 | - | L | retron-type RNA-directed DNA polymerase |
| RLPCCGM1_p0567 | - | - | 3-hydroxyalkanoate synthetase |
| RLPCCGM1_p0569 | - | - | hypothetical protein |
| RLPCCGM1_p0570 | - | - | monophenol monooxygenase |
| RLPCCGM1_p0571 | - | L | insertion sequence transposase |
| RLPCCGM1_p0573 | - | - | hypothetical protein |
| RLPCCGM1_p0574 | - | - | hypothetical protein |

| | | | |
|---|---|---|---|
| RLPCCGM1_p0575 | - | - | hypothetical protein |
| RLPCCGM1_p0597 | - | - | hypothetical protein |
| RLPCCGM1_p0599 | - | - | hypothetical protein |
| RLPCCGM1_p0602 | - | L | transposase |
| RLPCCGM1_p0603 | - | L | transposase |
| RLPCCGM1_p0612 | - | L | transposase |
| RLPCCGM1_p0613 | - | K | LysR family transcriptional regulator |
| RLPCCGM1_p0618 | - | Q | L-ascorbate oxidase |
| RLPCCGM1_p0627 | - | - | ABC transporter, permease protein |
| RLPCCGM1_p0634 | - | T | two-component sensor histidine kinase/response regulator hybrid protein (FixL) |
| RLPCCGM1_p0643 | - | P | cation transport P-type ATPase |
| RLPCCGM1_p0646 | - | - | hypothetical protein |
| RLPCCGM1_p0647 | - | - | hypothetical protein |
| RLPCCGM1_p0648 | - | - | hypothetical protein |
| RLPCCGM1_p0649 | - | - | hypothetical protein |
| RLPCCGM1_p0650 | - | O | ATPase |
| RLPCCGM1_p0665 | *nolL* | G | nodulation acetyltransferase |
| RLPCCGM1_p0674 | - | C | hypothetical protein |
| RLPCCGM1_p0677 | - | L | insertion sequence IS21 ATP-binding protein |
| RLPCCGM1_p0678 | - | L | transposase |
| RLPCCGM1_p0685 | *retA* | L | RNA-directed DNA polymerase RetA |
| RLPCCGM1_p0692 | *xylB* | C | aryl-alcohol dehydrogenase |
| RLPCCGM1_p0693 | - | G | deoxyribose-specific ABC transporter, permease protein |
| RLPCCGM1_p0706 | - | L | hypothetical protein |
| RLPCCGM1_p0712 | - | R | metallo-hydrolase |
| RLPCCGM1_p0718 | - | - | transposase |
| RLPCCGM1_p0759 | *nolO* | O | nodulation protein NolO |
| RLPCCGM1_p0768 | - | L | insertion sequence transposase protein |
| RLPCCGM1_p0769 | - | L | transposase |
| RLPCCGM1_p0775 | *cpxP* | Q | cytochrome P450 monooxygenase |
| RLPCCGM1_p0780 | - | L | hypothetical protein |
| RLPCCGM1_p0782 | - | L | hypothetical protein |
| RLPCCGM1_p0784 | - | - | hypothetical protein |
| RLPCCGM1_p0789 | - | - | hypothetical protein |
| RLPCCGM1_p0807 | - | L | transposase |
| RLPCCGM1_p0809 | - | L | transposase |
| RLPCCGM1_p0811 | - | L | insertion sequence transposase protein, IS21/IS408/IS1162 family |
| RLPCCGM1_p0812 | - | L | IS21 family insertion sequence ATP-binding protein |
| RLPCCGM1_p0818 | - | - | hypothetical protein |
| RLPCCGM1_p0849 | *ooxA* | R | opine oxidase subunit A |
| RLPCCGM1_p0851 | *ooxB* | E | opine oxidase subunit B |
| RLPCCGM1_p0858 | - | - | 2-haloacid dehalogenase protein |
| RLPCCGM1_p0884 | - | O | heat shock protein 60 family chaperone GroEL |
| RLPCCGM1_p0889 | - | R | multimeric flavodoxin WrbA |
| RLPCCGM1_p0929 | - | S | hypothetical protein |
| RLPCCGM1_p0930 | - | S | hypothetical protein |
| RLPCCGM1_p0937 | - | - | hypothetical protein |
| RLPCCGM1_p1014 | - | H | gamma-aminobutyratealpha-ketoglutarate aminotransferase |
| RLPCCGM1_p1015 | - | H | gamma-aminobutyratealpha-ketoglutarate aminotransferase |
| RLPCCGM1_p1018 | - | - | hypothetical protein |
| RLPCCGM1_p1148 | - | - | hypothetical protein |
| RLPCCGM1_p1187 | - | - | hypothetical protein |
| RLPCCGM1_p1188 | - | - | hypothetical protein |

| | | | |
|---|---|---|---|
| RLPCCGM1_p1320 | *amiF* | R | formamidase |
| RLPCCGM1_p1338 | - | - | hypothetical protein |
| RLPCCGM1_p1349 | *cheR* | N | chemotaxis methyltransferase |
| RLPCCGM1_p1350 | *cheB* | N | chemotaxis protein-glutamate methylesterase |
| RLPCCGM1_p1352 | - | T | two-component response regulator protein |
| RLPCCGM1_p1360 | - | T | two-component sensor histidine kinase |
| RLPCCGM1_p1367 | - | - | hypothetical protein |
| RLPCCGM1_p1369 | - | S | hypothetical protein |
| RLPCCGM1_p1380 | - | S | hypothetical protein |
| RLPCCGM1_p1393 | - | L | hypothetical protein |
| RLPCCGM1_p1417 | - | - | hypothetical protein |
| RLPCCGM1_p1486 | *vbsA* | J | N6-hydroxylysine O-acetyltransferase |
| RLPCCGM1_p1514 | - | R | multi-sensor signal transduction multi-kinase |
| RLPCCGM1_p1524 | - | S | hypothetical protein |
| RLPCCGM1_p1525 | - | L | addiction module antitoxin |
| RLPCCGM1_p1526 | - | M | xylose isomerase |
| RLPCCGM1_p1527 | - | G | periplasmic binding protein/LacI transcriptional regulator |
| RLPCCGM1_p1530 | - | K | hypothetical protein |
| RLPCCGM1_p1532 | - | G | fucose mutarotase |
| RLPCCGM1_p1535 | - | Q | cytochrome P450-pinF2, plant-inducible |
| RLPCCGM1_p1539 | - | - | hypothetical protein |
| RLPCCGM1_p1540 | *syrB* | - | syrB regulator |
| RLPCCGM1_p1541 | - | - | hypothetical protein |
| RLPCCGM1_p1542 | - | - | hypothetical protein |
| RLPCCGM1_p1545 | - | E | hypothetical protein |
| RLPCCGM1_p1550 | - | G | extracellular solute-binding protein family 1 |
| RLPCCGM1_p1554 | - | - | inulin fructotransferase |
| RLPCCGM1_p1556 | - | R | hypothetical protein |
| RLPCCGM1_p1558 | *stcd* | C | oxidoreductase, FMN-binding protein |
| RLPCCGM1_p1562 | - | P | ABC transporter substrate-binding protein |
| RLPCCGM1_p1564 | - | P | binding-protein-dependent transport systems inner membrane component |
| RLPCCGM1_p1566 | - | H | hypothetical protein |
| RLPCCGM1_p1567 | - | T | two-component response regulator protein |
| RLPCCGM1_p1568 | - | T | two-component sensor histidine kinase |
| RLPCCGM1_p1569 | - | G | sugar ABC transporter substrate-binding protein |
| RLPCCGM1_p1571 | - | G | inner-membrane translocator |
| RLPCCGM1_p1579 | - | G | extracellular solute-binding protein family 1 |
| RLPCCGM1_p1581 | - | - | hypothetical protein |
| RLPCCGM1_p1582 | - | S | hypothetical protein |
| RLPCCGM1_p1584 | - | Q | hypothetical protein |
| RLPCCGM1_p1588 | - | - | hypothetical protein |
| RLPCCGM1_p1592 | - | S | hypothetical protein |
| RLPCCGM1_p1593 | - | I | bifunctional 3-hydroxyacyl-CoA dehydrogenase/thioesterase |
| RLPCCGM1_p1594 | - | I | acyl-CoA dehydrogenase |
| RLPCCGM1_p1595 | - | C | acyl-CoA synthetase |
| RLPCCGM1_p1601 | - | K | Cro/CI family transcriptional regulator |
| RLPCCGM1_p1603 | - | R | peptidase |
| RLPCCGM1_p1607 | - | R | hypothetical protein |
| RLPCCGM1_p1617 | - | G | sugar ABC transporter substrate-binding protein |
| RLPCCGM1_p1618 | - | - | hypothetical protein |
| RLPCCGM1_p1619 | - | T | diguanylate cyclase |
| RLPCCGM1_p1621 | - | E | sodium/glucose cotransporter |
| RLPCCGM1_p1624 | - | T | two-component sensor histidine kinase |

| | | | |
|---|---|---|---|
| RLPCCGM1_p1630 | - | E | homocysteine S-methyltransferase |
| RLPCCGM1_p1631 | - | K | MarR family transcriptional regulator |
| RLPCCGM1_p1632 | - | P | chloride channel protein |
| RLPCCGM1_p1634 | - | P | aliphatic sulfonate ABC transporter substrate-binding protein |
| RLPCCGM1_p1638 | - | G | sugar ABC transporter substrate-binding protein |
| RLPCCGM1_p1646 | - | - | hypothetical protein |
| RLPCCGM1_p1647 | - | G | sugar phosphate isomerase/epimerase |
| RLPCCGM1_p1650 | *iolH* | G | inositol utilization protein H HolH |
| RLPCCGM1_p1656 | *menG* | H | S-adenosylmethionine 2-demethylmenaquinone methyltransferase |
| RLPCCGM1_p1665 | - | E | hypothetical protein |
| RLPCCGM1_p1666 | - | E | hypothetical protein |
| RLPCCGM1_p1667 | - | - | hypothetical protein |
| RLPCCGM1_p1670 | - | K | transcriptional regulator, CopG family |
| RLPCCGM1_p1672 | - | G | myo-inositol catabolism protein |
| RLPCCGM1_p1677 | - | E | oxidoreductase |
| RLPCCGM1_p1692 | - | - | hypothetical protein |
| RLPCCGM1_p1716 | - | M | hypothetical protein |
| RLPCCGM1_p1723 | - | R | hypothetical protein |
| RLPCCGM1_p1724 | - | - | hypothetical protein |
| RLPCCGM1_p1725 | - | - | hypothetical protein |
| RLPCCGM1_p1726 | - | - | hypothetical protein |
| RLPCCGM1_p1727 | - | T | membrane protein |
| RLPCCGM1_p1728 | - | - | hypothetical protein |
| RLPCCGM1_p1729 | - | - | hypothetical protein |
| RLPCCGM1_p1730 | - | S | hypothetical protein |
| RLPCCGM1_p1731 | - | P | FAD-dependent pyridine nucleotide-disulfide oxidoreductase |
| RLPCCGM1_p1733 | - | S | hypothetical protein |
| RLPCCGM1_p1734 | - | - | hypothetical protein |
| RLPCCGM1_p1736 | - | - | ABC transporter integral membrane protein |
| RLPCCGM1_p1739 | - | - | lipoprotein |
| RLPCCGM1_p1741 | - | T | sensory histidine kinase QseC |
| RLPCCGM1_p1742 | - | K | phosphosugar isomerase |
| RLPCCGM1_p1743 | - | S | transmembrane protein |
| RLPCCGM1_p1744 | - | T | hypothetical protein |
| RLPCCGM1_p1746 | - | - | permeases of the drug/metabolite transporter (DMT) superfamily |
| RLPCCGM1_p1747 | - | - | hypothetical protein |
| RLPCCGM1_p1748 | - | R | carboxylesterase |
| RLPCCGM1_p1749 | - | - | fatty acid desaturase |
| RLPCCGM1_p1750 | - | G | hypothetical protein |
| RLPCCGM1_p1751 | - | - | hypothetical protein |
| RLPCCGM1_p1753 | - | E | phosphoserine phosphatase |
| RLPCCGM1_p1758 | *rbsB* | G | ribose ABC transporter, substrate-binding protein |
| RLPCCGM1_p1762 | - | K | GreA/GreB family elongation factor |
| RLPCCGM1_p1763 | *rnk* | K | regulator of nucleoside diphosphate kinase |
| RLPCCGM1_p1768 | - | - | cell division protein FtsH |
| RLPCCGM1_p1775 | - | - | hypothetical protein |
| RLPCCGM1_p1788 | - | - | hypothetical protein |
| RLPCCGM1_p1789 | - | - | hypothetical protein |
| RLPCCGM1_p1827 | - | - | hypothetical protein |
| RLPCCGM1_p1838 | - | L | IS3 family transposase |
| RLPCCGM1_p1846 | - | - | hypothetical protein |
| RLPCCGM1_p1857 | *cheR* | N | chemotaxis methyltransferase |
| RLPCCGM1_p1858 | *cheB* | N | chemotaxis protein-glutamate methylesterase |
| RLPCCGM1_p1860 | - | T | two-component response regulator protein |

| | | | |
|---|---|---|---|
| RLPCCGM1_p1868 | - | T | two-component sensor histidine kinase |
| RLPCCGM1_p1875 | - | - | hypothetical protein |
| RLPCCGM1_p1877 | - | S | hypothetical protein |
| RLPCCGM1_p1888 | - | S | hypothetical protein |
| RLPCCGM1_p1901 | - | L | insertion sequence transposase |
| RLPCCGM1_p1942 | - | - | transmembrane protein |
| RLPCCGM1_p1946 | - | - | hypothetical protein |
| RLPCCGM1_p1949 | - | G | extracellular solute-binding protein family 1 |
| RLPCCGM1_p1971 | - | - | hypothetical protein |
| RLPCCGM1_p2011 | - | Q | isochorismatase |
| RLPCCGM1_p2022 | - | H | dimethylmenaquinone methyltransferase |
| RLPCCGM1_p2023 | - | K | IclR family transcriptional regulator |
| RLPCCGM1_p2024 | - | E | dipeptide-binding ABC transporter |
| RLPCCGM1_p2029 | - | - | hypothetical protein |
| RLPCCGM1_p2030 | - | G | hypothetical protein |
| RLPCCGM1_p2032 | - | T | sensory box/GGDEF family protein |
| RLPCCGM1_p2033 | - | - | hypothetical protein |
| RLPCCGM1_p2035 | *cpaB* | U | CpaB pilus assembly protein |
| RLPCCGM1_p2038 | - | - | hypothetical protein |
| RLPCCGM1_p2039 | - | U | hypothetical protein |
| RLPCCGM1_p2040 | - | U | hypothetical protein |
| RLPCCGM1_p2041 | - | U | pilus assembly protein |
| RLPCCGM1_p2046 | - | O | AAA ATPase |
| RLPCCGM1_p2047 | - | - | hypothetical protein |
| RLPCCGM1_p2048 | - | U | hypothetical protein |
| RLPCCGM1_p2049 | *nfeD* | O | nodulation competitiveness protein nfeD |
| RLPCCGM1_p2050 | - | O | hypothetical protein |
| RLPCCGM1_p2058 | - | K | AraC family transcriptional regulator |
| RLPCCGM1_p2060 | - | K | GCN5 N-acetyltransferase |
| RLPCCGM1_p2067 | - | I | MaoC dehydratase family protein |
| RLPCCGM1_p2070 | *cpxA* | T | sensor protein CpxA |
| RLPCCGM1_p2072 | - | M | efflux cation transporter protein |
| RLPCCGM1_p2074 | - | - | hypothetical protein |
| RLPCCGM1_p2076 | - | - | hypothetical protein |
| RLPCCGM1_p2077 | - | G | hypothetical protein |
| RLPCCGM1_p2078 | - | G | 5-deoxy-glucuronate isomerase |
| RLPCCGM1_p2088 | - | - | Leu/Ile/Val-binding protein |
| RLPCCGM1_p2090 | *livH* | E | branched-chain amino acid ABC transporter |
| RLPCCGM1_p2092 | - | C | hypothetical protein |
| RLPCCGM1_p2097 | - | - | alpha-L-rhamnosidase |
| RLPCCGM1_p2098 | - | G | extracellular solute-binding protein family 1 |
| RLPCCGM1_p2104 | *gmhA* | G | phosphoheptose isomerase |
| RLPCCGM1_p2105 | - | M | glycosyl transferase family 2 |
| RLPCCGM1_p2106 | - | - | hypothetical protein |
| RLPCCGM1_p2107 | - | - | hypothetical protein |
| RLPCCGM1_p2109 | - | E | histidinol-phosphate phosphatase family protein |
| RLPCCGM1_p2110 | - | R | GHMP kinase |
| RLPCCGM1_p2111 | - | M | NAD-dependent epimerase/dehydratase |
| RLPCCGM1_p2112 | - | - | FAD dependent oxidoreductase |
| RLPCCGM1_p2113 | - | P | hypothetical protein |
| RLPCCGM1_p2119 | - | - | hypothetical protein |
| RLPCCGM1_p2125 | - | L | hypothetical protein |
| RLPCCGM1_p2127 | - | S | hypothetical protein |
| RLPCCGM1_p2128 | *tauA* | P | taurine uptake ABC transporter substrate-binding protein |

| | | | |
|---|---|---|---|
| RLPCCGM1_p2132 | - | - | hypothetical protein |
| RLPCCGM1_p2137 | - | Q | 2-hydroxychromene-2-carboxylate isomerase |
| RLPCCGM1_p2138 | - | C | oxidoreductase |
| RLPCCGM1_p2140 | - | E | periplasmic binding protein |
| RLPCCGM1_p2141 | - | E | amino acid ABC transporter substrate-binding protein |
| RLPCCGM1_p2143 | - | K | acetyltransferase |
| RLPCCGM1_p2146 | - | C | oxidoreductase |
| RLPCCGM1_p2149 | - | - | hypothetical protein |
| RLPCCGM1_p2154 | - | K | hypothetical protein |
| RLPCCGM1_p2155 | *otsB* | G | trehalose-phosphatase |
| RLPCCGM1_p2159 | - | G | sugar ABC transporter permease |
| RLPCCGM1_p2163 | - | G | 4-hydroxyphenylpyruvate dioxygenase |
| RLPCCGM1_p2165 | - | G | extracytoplasmic solute receptor of TRAP-transport system |
| RLPCCGM1_p2167 | - | G | small transmembrane subunit, TRAP-transport system |
| RLPCCGM1_p2169 | - | T | two-component sensor histidine kinase |
| RLPCCGM1_p2171 | - | - | hypothetical protein |
| RLPCCGM1_p2176 | - | L | hypothetical protein |
| RLPCCGM1_p2177 | - | R | general stress protein 26 |
| RLPCCGM1_p2180 | - | S | UDP-galactose-lipid carrier transferase |
| RLPCCGM1_p2182 | - | L | hypothetical protein |
| RLPCCGM1_p2183 | - | - | hypothetical protein |

***Sinorhizobium americanum* CCGM7**

| ID | Name | COG | Function |
|---|---|---|---|
| SAMCCGM7_Ch0113 | - | - | hypothetical protein |
| SAMCCGM7_Ch0198 | - | R | glutamate synthase [NADPH] large chain |
| SAMCCGM7_Ch0278 | *ftsK* | D | cell division protein FtsK |
| SAMCCGM7_Ch0823 | - | - | hypothetical protein |
| SAMCCGM7_Ch0897 | - | E | transporter, LysE family |
| SAMCCGM7_Ch0913 | - | - | hypothetical protein |
| SAMCCGM7_Ch0937 | - | R | acetyltransferase |
| SAMCCGM7_Ch0973 | - | - | hypothetical protein |
| SAMCCGM7_Ch1414 | *smf* | L | DNA processing protein DprA |
| SAMCCGM7_Ch1426 | - | - | hypothetical protein |
| SAMCCGM7_Ch1804 | - | S | rhamnosyltransferase family protein |
| SAMCCGM7_Ch2020 | - | - | hypothetical protein |
| SAMCCGM7_Ch2023 | - | - | hypothetical protein |
| SAMCCGM7_Ch2148 | - | R | secretion activator protein |
| SAMCCGM7_Ch2150 | - | - | hypothetical protein |
| SAMCCGM7_Ch2151 | - | R | methyltransferase |
| SAMCCGM7_Ch2207 | - | - | hypothetical protein |
| SAMCCGM7_Ch2217 | - | - | hypothetical protein |
| SAMCCGM7_Ch2238 | - | R | SAM-dependent methyltransferase protein |
| SAMCCGM7_Ch2240 | - | R | aldo/keto reductase |
| SAMCCGM7_Ch2248 | - | - | hypothetical protein |
| SAMCCGM7_Ch2256 | - | Q | 2-hydroxychromene-2-carboxylate isomerase protein |
| SAMCCGM7_Ch2365 | - | E | N-methyl-l-tryptophan oxidase |
| SAMCCGM7_Ch2642 | - | - | hypothetical protein |
| SAMCCGM7_Ch2702 | - | L | DNA methylase |
| SAMCCGM7_Ch2825 | - | N | type IV pilus assembly protein |
| SAMCCGM7_Ch2829 | - | O | GTP-binding protein EngA |
| SAMCCGM7_Ch2975 | - | R | multidrug transporter |
| SAMCCGM7_Ch3017 | - | - | hypothetical protein |
| SAMCCGM7_Ch3097 | - | R | NADH dehydrogenase |

| | | | |
|---|---|---|---|
| SAMCCGM7_Ch3363 | - | C | cytochrome c peroxidase |
| SAMCCGM7_Ch3364 | - | C | cytochrome c peroxidase |
| SAMCCGM7_Ch3409 | - | - | hypothetical protein |
| SAMCCGM7_Ch3413 | - | V | type I restriction enzyme MjaXP M protein |
| SAMCCGM7_Ch3414 | - | V | restriction endonuclease S subunit |
| SAMCCGM7_Ch3415 | - | V | type I restriction enzyme hindVIIP R protein |
| SAMCCGM7_Ch3416 | - | R | metal-dependent hydrolase |
| SAMCCGM7_Ch3419 | - | - | hypothetical protein |
| SAMCCGM7_Ch3420 | - | - | hypothetical protein |
| SAMCCGM7_Ch3421 | - | - | hypothetical protein |
| SAMCCGM7_Ch3424 | - | - | hypothetical protein |
| SAMCCGM7_Ch3426 | - | L | integrase family protein |
| SAMCCGM7_Ch3465 | - | R | AAA ATPase |
| SAMCCGM7_Ch3466 | - | R | ATPase associated with various cellular activity, AAA_5 |
| SAMCCGM7_Ch3499 | *ligT* | J | 2'-5' RNA ligase |
| SAMCCGM7_Ch3542 | - | - | hypothetical protein |
| SAMCCGM7_Ch3594 | - | - | hypothetical protein |
| SAMCCGM7_pA0005 | - | - | hypothetical protein |
| SAMCCGM7_pA0011 | - | L | hypothetical protein |
| SAMCCGM7_pA0012 | - | L | hypothetical protein |
| SAMCCGM7_pA0013 | - | - | hypothetical protein |
| SAMCCGM7_pA0015 | - | J | hypothetical protein |
| SAMCCGM7_pA0020 | - | X | transposase |
| SAMCCGM7_pA0021 | - | L | transposase |
| SAMCCGM7_pA0023 | - | - | hypothetical protein |
| SAMCCGM7_pA0025 | - | - | hypothetical protein |
| SAMCCGM7_pA0026 | - | - | hypothetical protein |
| SAMCCGM7_pA0037 | *impM* | S | phosphatase ImpM |
| SAMCCGM7_pA0052 | - | - | hypothetical protein |
| SAMCCGM7_pA0057 | - | - | hypothetical protein |
| SAMCCGM7_pA0070 | *parA* | N | ParA protein, plasmid partitioning protein |
| SAMCCGM7_pA0079 | - | R | NAD(P)H dehydrogenase |
| SAMCCGM7_pA0081 | - | H | pyridoxamine 5'-phosphate oxidase |
| SAMCCGM7_pA0121 | *aacC* | V | aminoglycoside N(3')-acetyltransferase III |
| SAMCCGM7_pA0131 | - | E | sarcosine dehydrogenase |
| SAMCCGM7_pA0133 | - | E | saccharopine dehydrogenase |
| SAMCCGM7_pA0134 | - | I | choline kinase |
| SAMCCGM7_pA0136 | - | F | hydroxymethylpyrimidine transporter CytX |
| SAMCCGM7_pA0137 | - | K | TetR family transcriptional regulator |
| SAMCCGM7_pA0140 | - | R | 3-keto-5-aminohexanoate cleavage enzyme |
| SAMCCGM7_pA0141 | - | R | saccharopine dehydrogenase |
| SAMCCGM7_pA0142 | *lpsI* | I | succinyl-CoA:3-ketoacid coenzyme A transferase subunit A |
| SAMCCGM7_pA0143 | *lpsJ* | I | succinyl-CoA:3-ketoacid coenzyme A transferase subunit B |
| SAMCCGM7_pA0182 | - | - | hypothetical protein |
| SAMCCGM7_pA0234 | - | R | short-chain dehydrogenase/reductase SDR |
| SAMCCGM7_pA0235 | - | K | TetR family transcriptional regulator |
| SAMCCGM7_pA0286 | - | - | hypothetical protein |
| SAMCCGM7_pA0294 | *fabG* | I | 3-oxoacyl-[acyl-carrier protein] reductase |
| SAMCCGM7_pA0295 | - | J | cytidine/deoxycytidylate deaminase family protein |
| SAMCCGM7_pA0297 | *ubiE* | H | ubiquinone/menaquinone biosynthesis methyltransferase UbiE |
| SAMCCGM7_pA0298 | - | E | L-asparaginase |
| SAMCCGM7_pA0301 | - | - | hypothetical protein |
| SAMCCGM7_pA0302 | - | P | alkaline phosphatase |
| SAMCCGM7_pA0303 | - | K | two component system response regulator |

| | | | |
|---|---|---|---|
| SAMCCGM7_pA0304 | - | K | hypothetical protein |
| SAMCCGM7_pA0311 | - | K | transcriptional regulator, AraC family protein |
| SAMCCGM7_pA0315 | - | K | transcriptional regulator,AraC family |
| SAMCCGM7_pA0316 | - | O | hypothetical protein |
| SAMCCGM7_pA0318 | - | C | NADPH:quinone oxidoreductase |
| SAMCCGM7_pA0353 | *cya* | K | adenylate cyclase |
| SAMCCGM7_pA0354 | *cya* | K | adenylate cyclase |
| SAMCCGM7_pA0357 | - | U | T1SS secreted agglutinin RTX |
| SAMCCGM7_pA0368 | - | R | plasmid stabilization protein |
| SAMCCGM7_pA0371 | - | D | ParB domain protein nuclease |
| SAMCCGM7_pB0011 | - | - | hypothetical protein |
| SAMCCGM7_pB0012 | - | - | hypothetical protein |
| SAMCCGM7_pB0023 | - | X | ISSod11, transposase |
| SAMCCGM7_pB0029 | *traH* | L | conjugal transfer protein TraH |
| SAMCCGM7_pB0066 | *nodS* | Q | nodulation protein NodS |
| SAMCCGM7_pB0096 | - | - | hypothetical protein |
| SAMCCGM7_pB0097 | - | - | hypothetical protein |
| SAMCCGM7_pB0098 | - | R | extracellular protease |
| SAMCCGM7_pB0101 | - | R | serine protease, subtilase family |
| SAMCCGM7_pB0102 | - | - | hypothetical protein |
| SAMCCGM7_pB0103 | - | - | hypothetical protein |
| SAMCCGM7_pB0105 | - | - | hypothetical protein |
| SAMCCGM7_pB0116 | *rpiR* | K | HTH-type transcriptional regulator RpiR |
| SAMCCGM7_pB0140 | - | X | transposase |
| SAMCCGM7_pB0141 | - | X | transposase |
| SAMCCGM7_pB0146 | - | - | hypothetical protein |
| SAMCCGM7_pB0172 | - | X | transposase for insertion sequence element IS1081 |
| SAMCCGM7_pB0175 | - | X | transposase |
| SAMCCGM7_pB0176 | - | X | transposase |
| SAMCCGM7_pB0181 | - | X | transposase |
| SAMCCGM7_pB0183 | - | X | transposase |
| SAMCCGM7_pB0185 | - | X | transposase |
| SAMCCGM7_pB0187 | - | X | transposase |
| SAMCCGM7_pB0198 | - | X | transposase for insertion sequence NGRIS-18a |
| SAMCCGM7_pB0201 | *murE* | M | UDP-N-acetylmuramoyl-L-alanyl-D-glutamate--2, 6-diaminopimelate ligase |
| SAMCCGM7_pB0203 | - | E | binding-protein-dependent transport system inner membrane protein |
| SAMCCGM7_pB0205 | - | R | succinate dehydrogenase subunit |
| SAMCCGM7_pB0209 | - | K | IclR family transcriptional regulator |
| SAMCCGM7_pB0210 | - | X | integrase/recombinase y4rC |
| SAMCCGM7_pB0213 | *ddpA* | E | D,D-dipeptide-binding periplasmic protein DdpA |
| SAMCCGM7_pB0220 | - | M | peptidase M14 |
| SAMCCGM7_pB0222 | - | X | transposase |
| SAMCCGM7_pB0226 | - | X | transposase |
| SAMCCGM7_pB0228 | - | X | transposase |
| SAMCCGM7_pB0232 | - | X | transposase |
| SAMCCGM7_pB0236 | *ocd* | E | ornithine cyclodeaminase 1 |
| SAMCCGM7_pB0238 | - | E | D-serine deaminase |
| SAMCCGM7_pB0240 | *argE* | E | acetylornithine deacetylase |
| SAMCCGM7_pB0243 | - | L | transposase number 4 for insertion sequence NGRIS-4e |
| SAMCCGM7_pB0248 | - | R | hypothetical protein |
| SAMCCGM7_pB0249 | - | H | hypothetical protein |
| SAMCCGM7_pB0250 | - | - | hypothetical protein |
| SAMCCGM7_pB0263 | - | E | amino acid ABC transporter permease protein |

| | | | |
|---|---|---|---|
| SAMCCGM7_pB0267 | - | - | hypothetical protein |
| SAMCCGM7_pB0268 | - | J | archaeosine tRNA-ribosyltransferase type 5 |
| SAMCCGM7_pB0269 | - | - | hypothetical protein |
| SAMCCGM7_pB0270 | - | R | queuosine Biosynthesis QueE Radical SAM |
| SAMCCGM7_pB0275 | - | - | hypothetical protein |
| SAMCCGM7_pB0277 | - | - | hypothetical protein |
| SAMCCGM7_pB0278 | - | - | hypothetical protein |
| SAMCCGM7_pB0283 | - | H | monooxygenase |
| SAMCCGM7_pB0288 | - | X | transposase |
| SAMCCGM7_pB0294 | *npt* | H | 4'-phosphopantetheinyl transferase Npt |
| SAMCCGM7_pB0301 | - | X | transposase |
| SAMCCGM7_pB0321 | - | R | transcriptional regulator, AraC family |
| SAMCCGM7_pB0365 | *hupB* | C | uptake hydrogenase large subunit |
| SAMCCGM7_pB0368 | *chrA* | P | chromate transport protein ChrA |
| SAMCCGM7_pB0375 | - | R | phosphatase |
| SAMCCGM7_pB0422 | - | E | polar amino acid ABC transporter, inner membrane subunit |
| SAMCCGM7_pB0436 | - | - | hypothetical protein |
| SAMCCGM7_pB0437 | - | K | transcriptional regulator, TetR family |
| SAMCCGM7_pB0448 | *pgl* | G | 6-phosphogluconolactonase |
| SAMCCGM7_pB0467 | - | - | hypothetical protein |
| SAMCCGM7_pC0022 | - | K | transcriptional regulator, TetR family |
| SAMCCGM7_pC0023 | - | G | cupin domain-containing protein |
| SAMCCGM7_pC0126 | - | X | phage DNA invertase |
| SAMCCGM7_pC0135 | - | - | hypothetical protein |
| SAMCCGM7_pC0136 | - | T | DNA helicase, UvrD/REP family |
| SAMCCGM7_pC0137 | *dnaK* | O | chaperone protein DnaK |
| SAMCCGM7_pC0138 | - | - | hypothetical protein |
| SAMCCGM7_pC0139 | - | - | hypothetical protein |
| SAMCCGM7_pC0144 | *catB* | M | muconate cycloisomerase 1-1 |
| SAMCCGM7_pC0146 | *catA* | Q | catechol 1,2-dioxygenase |
| SAMCCGM7_pC0147 | *cbdA* | P | 2-halobenzoate 1,2-dioxygenase large subunit |
| SAMCCGM7_pC0148 | *xylY* | Q | toluate 1,2-dioxygenase subunit beta |
| SAMCCGM7_pC0149 | *benC* | H | benzoate 1,2-dioxygenase electron transfer component |
| SAMCCGM7_pC0151 | *benK* | G | benzoate transport protein BenK |
| SAMCCGM7_pC0154 | - | E | creatinase |
| SAMCCGM7_pC0155 | *crnA* | H | creatinine amidohydrolase |
| SAMCCGM7_pC0157 | - | E | ABC transporter substrate-binding protein |
| SAMCCGM7_pC0162 | - | X | ISSod11, transposase |
| SAMCCGM7_pC0166 | *mntH* | P | manganese transport protein MntH |
| SAMCCGM7_pC0168 | - | - | hypothetical protein |
| SAMCCGM7_pC0169 | *phaZ* | Q | poly(3-hydroxyalkanoate) depolymerase C |
| SAMCCGM7_pC0170 | - | K | hypothetical protein |
| SAMCCGM7_pC0171 | *cheR* | N | chemotaxis protein methyltransferase CheR |
| SAMCCGM7_pC0174 | - | G | inosose isomerase |
| SAMCCGM7_pC0175 | - | - | hypothetical protein |
| SAMCCGM7_pC0181 | - | R | copper oxidase |
| SAMCCGM7_pC0182 | - | D | multicopper oxidase type 3 |
| SAMCCGM7_pC0183 | - | M | copper resistance-like lipoprotein |
| SAMCCGM7_pC0186 | - | - | hypothetical protein |
| SAMCCGM7_pC0190 | - | - | hypothetical protein |
| SAMCCGM7_pC0191 | - | R | surface polysaccharides/antigen |
| SAMCCGM7_pC0200 | - | X | integrase/recombinase y4rB |
| SAMCCGM7_pC0202 | - | X | transposase IS3/IS911 family protein |
| SAMCCGM7_pC0203 | - | - | hypothetical protein |

| | | | |
|---|---|---|---|
| SAMCCGM7_pC0204 | *hipA* | T | serine/threonine-protein kinase HipA |
| SAMCCGM7_pC0206 | - | R | diguanylate cyclase/phosphodiesterase (GGDEF & EAL domains) with PAS/PAC sensor(s) |
| SAMCCGM7_pC0210 | - | G | DMT superfamily inner membrane transporter protein |
| SAMCCGM7_pC0212 | - | O | hypothetical protein |
| SAMCCGM7_pC0214 | - | - | hypothetical protein |
| SAMCCGM7_pC0228 | - | K | transcriptional regulator |
| SAMCCGM7_pC0266 | - | H | hydrolase |
| SAMCCGM7_pC0272 | - | - | hypothetical protein |
| SAMCCGM7_pC0273 | - | T | TspO/MBR-related protein |
| SAMCCGM7_pC0275 | - | R | beta-Ig-H3/fasciclin |
| SAMCCGM7_pC0276 | - | G | transcriptional regulator |
| SAMCCGM7_pC0278 | - | H | hypothetical protein |
| SAMCCGM7_pC0300 | - | - | hypothetical protein |
| SAMCCGM7_pC0307 | - | S | copper metallochaperone |
| SAMCCGM7_pC0309 | - | P | copper binding protein |
| SAMCCGM7_pC0312 | - | S | DoxX family protein |
| SAMCCGM7_pC0314 | - | R | exoenzymes regulatory protein AepA precursor |
| SAMCCGM7_pC0316 | - | H | hydrolase |
| SAMCCGM7_pC0319 | - | V | endoribonuclease L-PSP |
| SAMCCGM7_pC0321 | *ftsH* | D | cell division protein FtsH |
| SAMCCGM7_pC0328 | - | K | transcriptional regulator |
| SAMCCGM7_pC0332 | - | R | NAD(P)H dehydrogenase, quinone family, Nqo1-like |
| SAMCCGM7_pC0336 | - | H | isochorismatase |
| SAMCCGM7_pC0343 | - | R | CHRD protein |
| SAMCCGM7_pC0346 | - | - | hypothetical protein |
| SAMCCGM7_pC0351 | - | S | hypothetical protein |
| SAMCCGM7_pC0352 | - | X | transposase |
| SAMCCGM7_pC0354 | - | X | transposase |
| SAMCCGM7_pC0357 | *prr* | C | gamma-aminobutyrate:alpha-ketoglutarate aminotransferase |
| SAMCCGM7_pC0363 | - | R | histone acetyltransferase HPA2 |
| SAMCCGM7_pC0364 | - | P | Na+/H+ antiporter protein |
| SAMCCGM7_pC0439 | - | E | glutamate synthase [NADPH] large chain |
| SAMCCGM7_pC0492 | *aprE* | O | subtilisin E |
| SAMCCGM7_pC0547 | - | - | hypothetical protein |
| SAMCCGM7_pC0548 | - | C | pseudoazurin |
| SAMCCGM7_pC0549 | - | K | sensory box sensor histidine kinase/response regulator |
| SAMCCGM7_pC0550 | - | K | HTH-type transcriptional regulator |
| SAMCCGM7_pC0557 | *hpaH* | Q | 2-oxo-hepta-3-ene-1,7-dioic acid hydratase |
| SAMCCGM7_pC0560 | - | G | sugar phosphate isomerases/epimerase |
| SAMCCGM7_pC0566 | - | M | 5-carboxymethyl-2-hydroxymuconate delta-isomerase |
| SAMCCGM7_pC0568 | - | R | oxidoreductase |
| SAMCCGM7_pC0573 | - | H | epoxide hydrolase |
| SAMCCGM7_pC0574 | - | H | epoxide hydrolase |
| SAMCCGM7_pC0585 | - | I | hydrolase |
| SAMCCGM7_pC0588 | - | S | membrane protein |
| SAMCCGM7_pC0589 | - | K | TetR family transcriptional regulator |
| SAMCCGM7_pC0602 | - | E | acetyltransferase |
| SAMCCGM7_pC0609 | *pdxA* | R | 4-hydroxythreonine-4-phosphate dehydrogenase |
| SAMCCGM7_pC0611 | - | S | inner membrane protein |
| SAMCCGM7_pC0631 | - | X | transposase |
| SAMCCGM7_pC0638 | - | E | metal-activated pyridoxal enzyme |
| SAMCCGM7_pC0645 | *thrA* | E | homoserine dehydrogenase |
| SAMCCGM7_pC0655 | - | E | acetyltransferase |

| | | | |
|---|---|---|---|
| SAMCCGM7_pC0657 | - | K | GntR family transcriptional regulator |
| SAMCCGM7_pC0659 | - | T | homoserine kinase type II |
| SAMCCGM7_pC0662 | - | T | multi-sensor signal transduction multi-kinase |
| SAMCCGM7_pC0673 | - | T | transmembrane sensory box GGDEF/EAL family protein |
| SAMCCGM7_pC0675 | - | Q | glyoxalase |
| SAMCCGM7_pC0676 | - | J | histone acetyltransferase HPA2 |
| SAMCCGM7_pC0686 | - | H | isochorismatase |
| SAMCCGM7_pC0690 | - | O | isoprenylcysteine carboxyl methyltransferase |
| SAMCCGM7_pC0693 | - | M | NodT, RND efflux system, outer membrane lipoprotein |
| SAMCCGM7_pC0694 | - | R | hypothetical protein |
| SAMCCGM7_pC0695 | - | M | MltA-interacting MipA family protein |
| SAMCCGM7_pC0697 | - | T | histidine kinase |
| SAMCCGM7_pC0698 | - | - | hypothetical protein |
| SAMCCGM7_pC0702 | - | R | beta-lactamase domain containing protein |
| SAMCCGM7_pC0713 | - | I | lipase |
| SAMCCGM7_pC0714 | - | H | signal peptide protein |
| SAMCCGM7_pC0721 | - | P | sulfonate ABC transporter substrate-binding protein |
| SAMCCGM7_pC0740 | - | - | hypothetical protein |
| SAMCCGM7_pC0741 | - | R | methyltransferase type 11 |
| SAMCCGM7_pC0745 | - | R | NAD(P)H dehydrogenase |
| SAMCCGM7_pC0747 | - | H | pyridoxamine 5'-phosphate oxidase |
| SAMCCGM7_pC0748 | *soxY* | S | sulfur oxidation protein SoxY |
| SAMCCGM7_pC0749 | *soxZ* | R | sulfur oxidation Z protein SoxZ |
| SAMCCGM7_pC0750 | - | C | cytochrome c553 |
| SAMCCGM7_pC0751 | *fccB* | I | sulfide dehydrogenase (flavocytochrome C) flavoprotein subunit |
| SAMCCGM7_pC0753 | - | C | hypothetical protein |
| SAMCCGM7_pC0754 | - | T | metallophosphoesterase |
| SAMCCGM7_pC0755 | - | C | lipoprotein |
| SAMCCGM7_pC0761 | *fixN* | C | cytochrome c oxidase subunit 1 |
| SAMCCGM7_pC0762 | *fixO* | C | cytochrome C oxidase mono-heme subunit/FixO |
| SAMCCGM7_pC0766 | - | P | heavy-metal-associated domain |
| SAMCCGM7_pC0769 | - | C | cytochrome c peroxidase |
| SAMCCGM7_pC0778 | - | G | major facilitator superfamily protein |
| SAMCCGM7_pC0781 | - | P | high-affinity nickel-transporter |
| SAMCCGM7_pC0782 | - | S | inner membrane protein |
| SAMCCGM7_pC0783 | - | G | hypothetical protein |
| SAMCCGM7_pC0787 | *nikA* | E | nickel-binding periplasmic protein NikA |
| SAMCCGM7_pC0788 | - | K | sensor histidine kinase domain-containing protein |
| SAMCCGM7_pC0798 | - | R | carboxymuconolactone decarboxylase |
| SAMCCGM7_pC0799 | - | K | LysR family transcriptional regulator |
| SAMCCGM7_pC0816 | - | - | hypothetical protein |
| SAMCCGM7_pC0817 | *ligD* | L | ATP-dependent DNA ligase |
| SAMCCGM7_pC0819 | *prtC* | V | serralysin C |
| SAMCCGM7_pC0821 | - | X | transposase |
| SAMCCGM7_pC0823 | *ligD* | L | ATP-dependent DNA ligase |
| SAMCCGM7_pC0826 | - | - | hypothetical protein |
| SAMCCGM7_pC0827 | - | - | hypothetical protein |
| SAMCCGM7_pC0831 | - | - | hypothetical protein |
| SAMCCGM7_pC0834 | *lpsZ* | M | lipopolysaccharide-processing protein LpsZ |
| SAMCCGM7_pC0835 | *rkpL* | M | polysaccharide biosynthesis protein UDP-4-dehydro-6-deoxy-2-acetamido-D-glucose 4-reductase |
| SAMCCGM7_pC0837 | *rkpN* | M | acylneuraminate cytidylyltransferase |
| SAMCCGM7_pC0838 | *rkpO* | M | polysaccharide biosynthesis protein |
| SAMCCGM7_pC0843 | *lpsZ* | M | lipopolysaccharide-processing protein LpsZ |

| | | | |
|---|---|---|---|
| SAMCCGM7_pC0847 | - | R | ATPase |
| SAMCCGM7_pC0848 | - | R | filamentation induced by cAMP protein Fic |
| SAMCCGM7_pC0850 | - | R | filamentation induced by cAMP protein Fic |
| SAMCCGM7_pC0872 | - | - | hypothetical protein |
| SAMCCGM7_pC0873 | *betA* | C | FAD dependent oxidoreductase |
| SAMCCGM7_pC0878 | - | X | transposase |
| SAMCCGM7_pC0923 | - | - | hypothetical protein |
| SAMCCGM7_pC1059 | - | - | hypothetical protein |
| SAMCCGM7_pC1084 | - | N | cellulose synthase catalytic subunit |
| SAMCCGM7_pC1137 | - | R | carbon-nitrogen family hydrolase |
| SAMCCGM7_pC1140 | - | I | transmembrane phospholipase protein |
| SAMCCGM7_pC1143 | - | - | hypothetical protein |
| SAMCCGM7_pC1232 | - | Q | hypothetical protein |
| SAMCCGM7_pC1266 | *lhgO* | G | L-2-hydroxyglutarate oxidase LhgO |
| SAMCCGM7_pC1297 | - | M | glycosyl transferase family protein |
| SAMCCGM7_pC1298 | - | M | mannosyl transferase |
| SAMCCGM7_pC1308 | - | D | CPSC/CAPB subfamily ATPase |
| SAMCCGM7_pC1323 | - | G | hypothetical protein |
| SAMCCGM7_pC1324 | - | U | HlyD family secretion protein |
| SAMCCGM7_pC1325 | - | - | hypothetical protein |
| SAMCCGM7_pC1327 | - | M | hypothetical protein |
| SAMCCGM7_pC1328 | - | - | hypothetical protein |
| SAMCCGM7_pC1329 | *pssL* | M | lipopolysaccharide biosynthesis protein wzxC |
| SAMCCGM7_pC1330 | - | G | glycosyl transferase family protein |
| SAMCCGM7_pC1331 | - | G | glycosyl transferase family protein |
| SAMCCGM7_pC1332 | *hemL* | H | glutamate-1-semialdehyde 2,1-aminomutase |
| SAMCCGM7_pC1333 | - | X | transposase for insertion sequence NGRIS-14b |
| SAMCCGM7_pC1335 | - | G | LmbE-like protein |
| SAMCCGM7_pC1339 | - | R | aminopeptidase |
| SAMCCGM7_pC1340 | - | M | NAD-dependent epimerase/dehydratase |
| SAMCCGM7_pC1363 | - | - | hypothetical protein |
| SAMCCGM7_pC1391 | - | E | ABC transporter binding protein |
| SAMCCGM7_pC1481 | - | - | hypothetical protein |
| SAMCCGM7_pC1485 | - | X | insertion sequence IS407 |
| SAMCCGM7_pC1548 | - | R | threonine efflux protein |
| SAMCCGM7_pC1575 | - | - | hypothetical protein |
| SAMCCGM7_pC1615 | - | L | ATP-dependent DNA ligase YkoU |
| SAMCCGM7_pC1709 | - | - | hypothetical protein |
| SAMCCGM7_pC1853 | - | K | AraC family transcriptional regulator |
| SAMCCGM7_pC1940 | - | O | small heat shock protein |
| SAMCCGM7_pC1945 | - | S | arsenate reductase |
| SAMCCGM7_pC1947 | - | O | ABC-type transport, permease protein |
| SAMCCGM7_pC1951 | *fabG* | I | 3-oxoacyl-ACP reductase |
| SAMCCGM7_pC1973 | - | J | cytidine/deoxycytidylate deaminase family protein |
| SAMCCGM7_pC2005 | - | T | HipA protein |
| SAMCCGM7_pC2006 | *spsA* | K | transcription regulator, symbiotic plasmid stability locus |
| SAMCCGM7_pC2008 | - | E | alpha/beta hydrolase family protein |
| SAMCCGM7_pC2009 | - | I | hypothetical protein |
| SAMCCGM7_pC2012 | - | R | transporter |
| SAMCCGM7_pC2013 | - | K | transcriptional regulator |
| SAMCCGM7_pC2014 | - | - | hypothetical protein |
| SAMCCGM7_pC2015 | - | O | cysteine protease |
| SAMCCGM7_pC2045 | - | X | ISSod11, transposase |
| SAMCCGM7_pC2047 | - | P | sulfatase |

| | | | |
|---|---|---|---|
| SAMCCGM7_pC2051 | - | G | sugar ABC transporter substrate-binding protein |
| SAMCCGM7_pC2053 | - | G | ABC transporter permease |
| SAMCCGM7_pC2055 | - | I | glycerophosphoryl diester phosphodiesterase |
| SAMCCGM7_pC2057 | - | - | hypothetical protein |
| SAMCCGM7_pC2060 | - | R | myo-inositol 2-dehydrogenase 1 |
| SAMCCGM7_pC2062 | - | R | short-chain oxidoreductase |
| SAMCCGM7_pC2064 | - | P | sulfur reduction protein DsrE |
| SAMCCGM7_pC2065 | - | R | beta-lactamase domain-containing protein |
| SAMCCGM7_pC2069 | - | K | transcriptional regulator |
| SAMCCGM7_pC2073 | - | X | ISSod11, transposase |
| SAMCCGM7_pC2074 | *glcF* | C | glycolate dehydrogenase, iron-sulfur subunit GlcF |
| SAMCCGM7_pC2082 | - | C | flavohemoprotein |
| SAMCCGM7_pC2084 | - | C | acetamidase |
| SAMCCGM7_pC2085 | - | K | helix-turn-helix-domain containing protein AraC type |
| SAMCCGM7_pC2086 | - | Q | alkyl sulfatase |
| SAMCCGM7_pC2092 | - | X | transposase for insertion sequence element |
| SAMCCGM7_pC2093 | - | - | hypothetical protein |
| SAMCCGM7_pC2094 | - | R | alpha/beta hydrolase |
| SAMCCGM7_pC2099 | - | K | ArsR family transcriptional regulator |
| SAMCCGM7_pC2102 | *ooxB* | E | opine oxidase subunit B |
| SAMCCGM7_pC2104 | *soxA* | I | sarcosine oxidase subunit alpha |
| SAMCCGM7_pC2106 | - | - | hypothetical protein |
