# Supplementary material for: Genomic studies of nitrogen-fixing rhizobial strains from Phaseolus vulgaris seeds and nodules
Source: BMC Genomics. 2016 Sep 6;17(1):711. doi: 10.1186/s12864-016-3053-z (PMC5011921; doi:10.1186/s12864-016-3053-z)
Supplement: Additional file 3: Table S2. — Average nucleotide identity (ANIm) among the genomes of selected Sinorhizobium and Rhizobium strains. (PDF 34 kb) [file 12864_2016_3053_MOESM3_ESM.pdf]

Supplementary Table 2. Average nucleotide identity (ANIm) among the genomes of selected *Sinorhizobium* and *Rhizobium* strains.

Sinorhizobium strains

		1	2	3	4	5	Genome size, Mb
CCGM7	1	-	98.3 (90.2)	88.4 (69.2)	88.7 (67.9)	88.7 (67.0)	6.9
CFNEI73	2	98.3 (91.5)	-	88.4 (69.4)	88.7 (67.8)	88.7 (67.2)	6.8
USDA257	3	88.5 (61.3)	88.4 (60.7)	-	89.8 (62.1)	89.1 (60.4)	7.6
HH103	4	88.8 (58.6)	88.8 (57.8)	90.1 (61.3)	-	92.2 (64.0)	7.8
NGR234	5	88.7 (66.7)	88.8 (66.4)	89.8 (72.8)	92.6 (78.9)	-	6.9

Rhizobium strains

		1	2	3	4	
CCGM1	1	-	97.1 (84.6)	98.0 (89.6)	90.5 (75.6)	6.9
CIAT652	2	97.1 (92.0)	-	97.3 (90.7)	90.4 (77.0)	6.4
CNPAF512	3	98.0 (93.6)	97.3 (86.9)	-	90.5 (76.1)	6.7
CFN42	4	90.5 (80.6)	90.4 (75.6)	90.5 (78.0)	-	6.5

Percentage of genome coverage in parenthesis.
