# Supplementary material for: Genomic studies of nitrogen-fixing rhizobial strains from Phaseolus vulgaris seeds and nodules
Source: BMC Genomics. 2016 Sep 6;17(1):711. doi: 10.1186/s12864-016-3053-z (PMC5011921; doi:10.1186/s12864-016-3053-z)
Supplement: Additional file 4: Figure S2. — Proteomes of R. phaseoli strains. A, CCGM1. B, CIAT652. Spots taken for analysis are encircled, in red, abundant proteins in the strain which were not visible in the other; in green, abundant proteins in the strain with counterpart in the other. Lines with arrows denote the direction of the 2D runs. (PDF 460 kb) [file 12864_2016_3053_MOESM4_ESM.pdf]

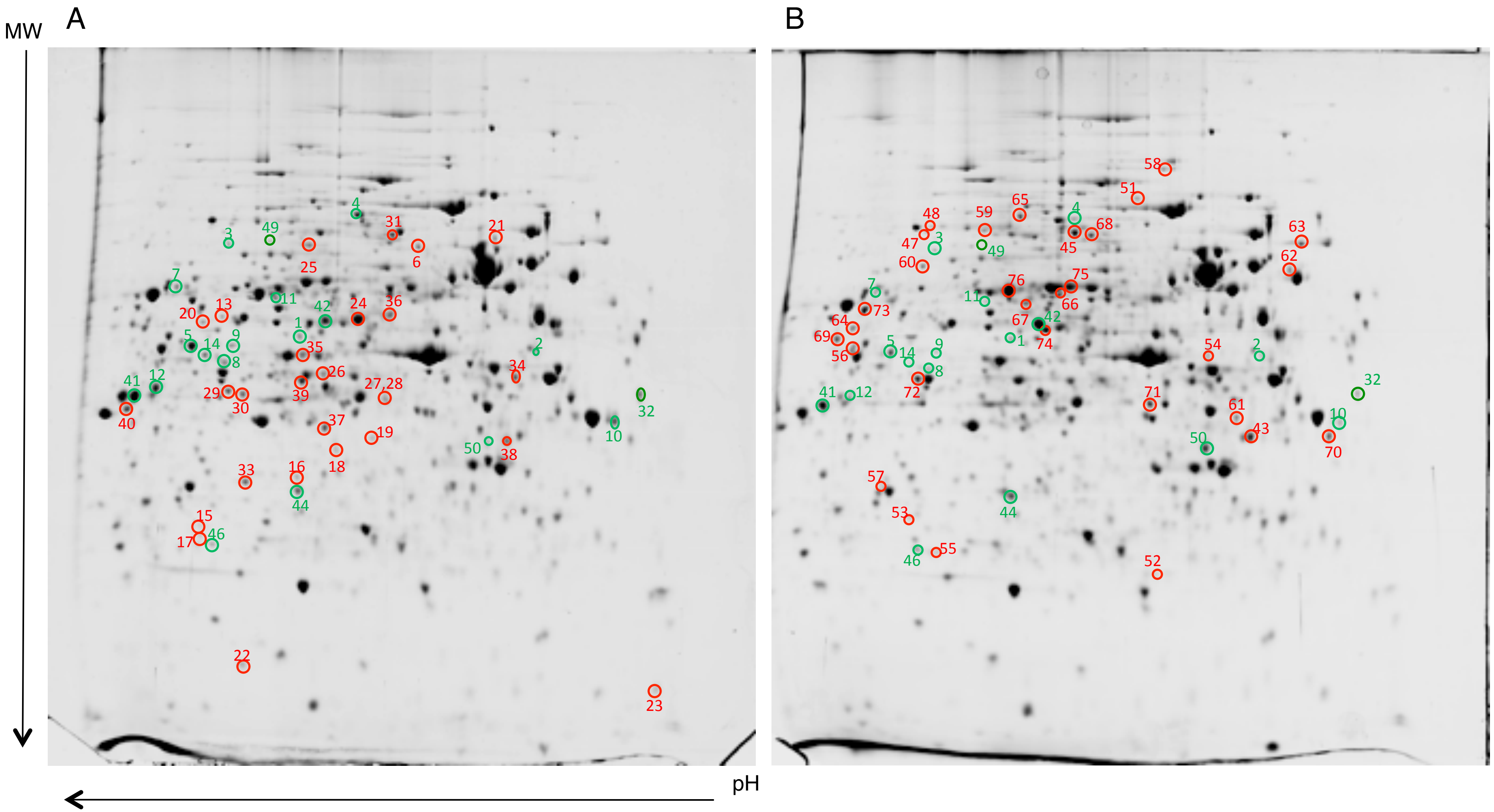

Supplementary Fig. 2. Proteomes of *R. phaseoli* strains. A, CCGM1. B, CIAT652. Spots taken for analysis are encircled, in red, abundant proteins in the strain which were not visible in the other; in green, abundant proteins in the strain with counterpart in the other. Lines with arrows denote the direction of the 2D runs.

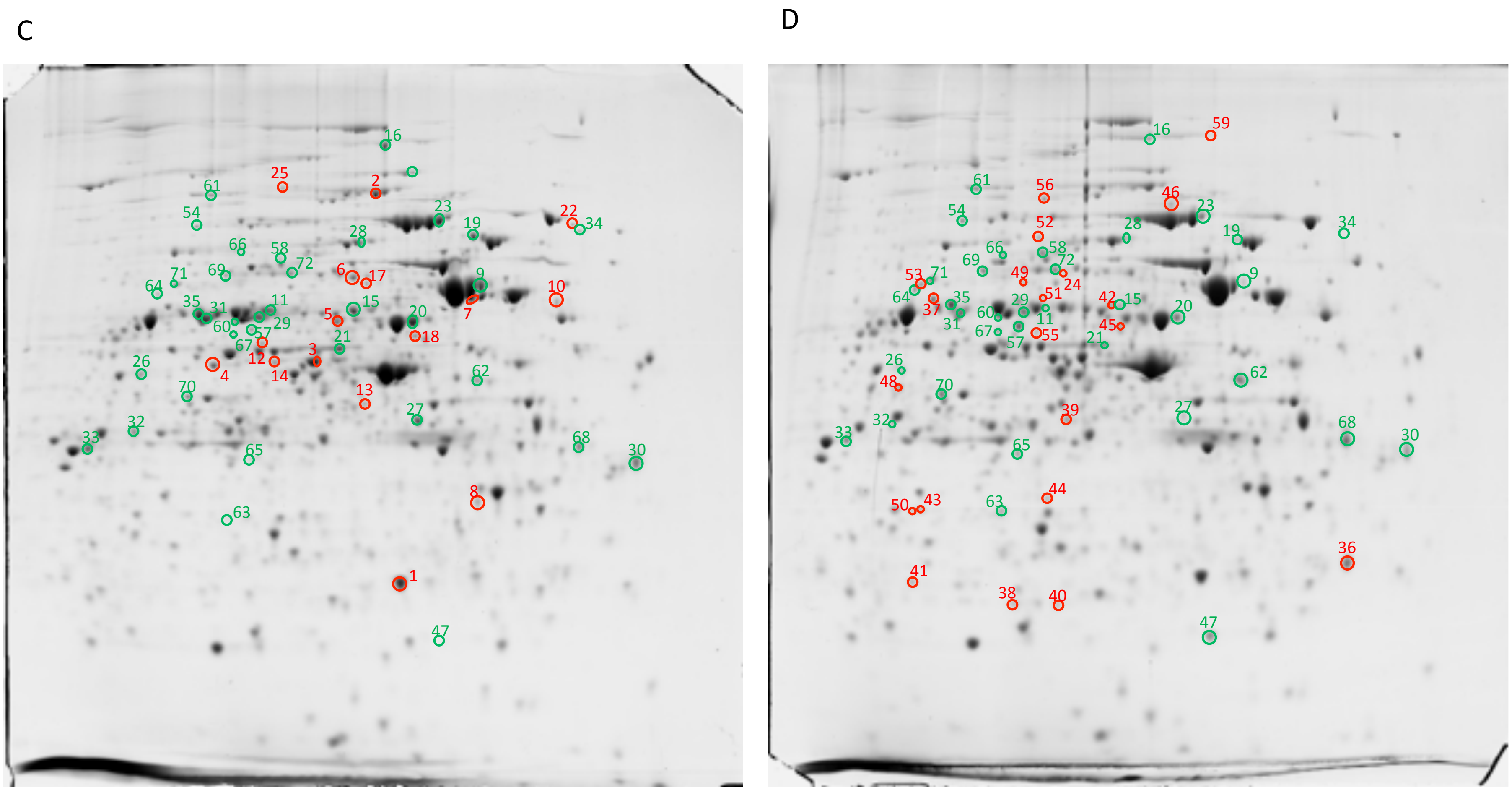

Supplementary Fig. 2. Proteomes of *S.americanum* strains. C, CCGM7. D, CFNEI73. Spots taken for analysis are encircled, in red, abundant proteins in the strain which were not visible in the other; in green, abundant proteins in the strain with counterpart in the other.
