# Supplementary material for: Genomic studies of nitrogen-fixing rhizobial strains from Phaseolus vulgaris seeds and nodules
Source: BMC Genomics. 2016 Sep 6;17(1):711. doi: 10.1186/s12864-016-3053-z (PMC5011921; doi:10.1186/s12864-016-3053-z)
Supplement: Additional file 5: Figure S3. — Metabolic reconstruction with BioCyc using the most abundant proteins of the strains detected in the proteome. A, Rhizobium phaseoli strains. Enzymes participating in metabolic pathways. Green boxes, the most abundant proteins from CCGM1 strain. Red boxes, the most abundant proteins from CIAT652 strain. B, Sinorhizobium americanum strains. Enzymes participating in metabolic pathways. Green boxes, the most abundant proteins from CCGM7 strain. Red boxes, the most abundant proteins from CFNEI73 strain. Brown were both coincided. The lists of proteins enable their use directly on the BioCyc page with the denoted organism. (−) denotes that no homolog was found in the strain. (PDF 355 kb) [file 12864_2016_3053_MOESM5_ESM.pdf]

A

Cellular Overview of *Rhizobium etli CIAT 652*

Pan left/right/up/down the entire diagram by holding the left mouse button, click on an object for more info, right-click (ctrl-click for Mac) for menu

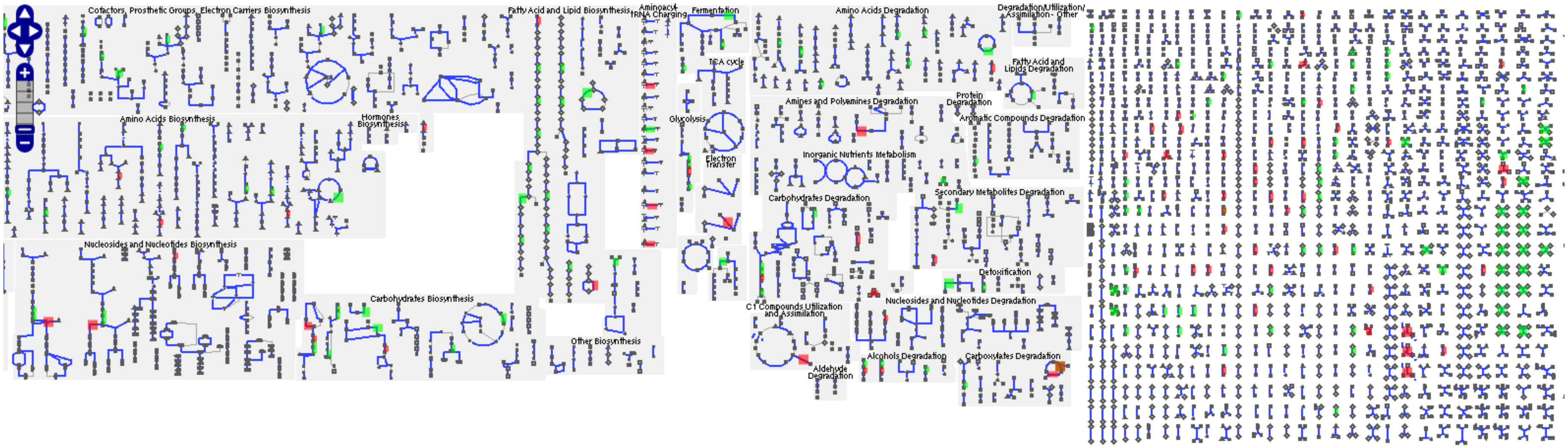

R. phaseoli **CCGM1.**

Spot no.	ID BIOCYC	Spot no.	ID BIOCYC
24	RHECIAT_CH0004168	17	RHECIAT_CH0000394
41	RHECIAT_CH0002030	18	RHECIAT_CH0004208
8	RHECIAT_PB0000381	35	RHECIAT_CH0000144
5	RHECIAT_CH0001858	1	RHECIAT_CH0002368
7	RHECIAT_CH0002358	15	RHECIAT_CH0003904
14	RHECIAT_PC0000303	13	RHECIAT_CH0003000
25	RHECIAT_CH0002930	33	RHECIAT_CH0003815
29	RHECIAT_CH0000591	10	RHECIAT_CH0000747
6	RHECIAT_CH0001854	21	RHECIAT_PC0000604
11	RHECIAT_CH0004399	22	RHECIAT_CH0002166
2	RHECIAT_CH0003742	31	RHECIAT_PC0000231
9	RHECIAT_CH0003793	4	RHECIAT_PC0000007
34	RHECIAT_CH0003050	16	RHECIAT_CH0001861
38	RHECIAT_CH0000536	27	RHECIAT_CH0003212
40	RHECIAT_CH0003737	28	RHECIAT_CH0000308
3	RHECIAT_CH0001005	30	RHECIAT_CH0003391
23	RHECIAT_CH0000005	19	RHECIAT_CH0000822
26	RHECIAT_CH0001030	37	RHECIAT_CH0003384
36	RHECIAT_CH0000032	12	RHECIAT_PB0000125
39	RHECIAT_CH0003694	20	RHECIAT_CH0000250
		32	RHECIAT_PC0000655

R. phaseoli **CIAT652.**

Spot no.	ID BIOCYC	Spot no.	ID BIOCYC
47	RHECIAT_PC0000173	59	RHECIAT_CH0002285
62	RHECIAT_CH0002031	65	RHECIAT_CH0000980
67	RHECIAT_CH0004343	66	RHECIAT_CH0002242
69	RHECIAT_CH0001680	57	RHECIAT_CH0001733
75	RHECIAT_CH0000039	74	RHECIAT_CH0003488
76	RHECIAT_CH0002032	46	RHECIAT_CH0001879
49	RHECIAT_PC0000423	52	RHECIAT_CH0001895
50	RHECIAT_CH0003659	63	RHECIAT_CH0002260
60	RHECIAT_CH0002150	70	RHECIAT_CH0004147
72	RHECIAT_CH0001992	71	RHECIAT_CH0002820
73	RHECIAT_CH0000879	56	RHECIAT_PC0000681
51	RHECIAT_CH0003979	43	RHECIAT_CH0004212
55	RHECIAT_CH0000213	44	RHECIAT_CH0004405
61	RHECIAT_CH0003393	68	RHECIAT_CH0002473
64	RHECIAT_CH0003248	45	RHECIAT_CH0004199
42	RHECIAT_CH0001955		
53	RHECIAT_CH0003282		
48	RHECIAT_PA0000110		
54	RHECIAT_CH0004024		
58	RHECIAT_CH0001897		

B

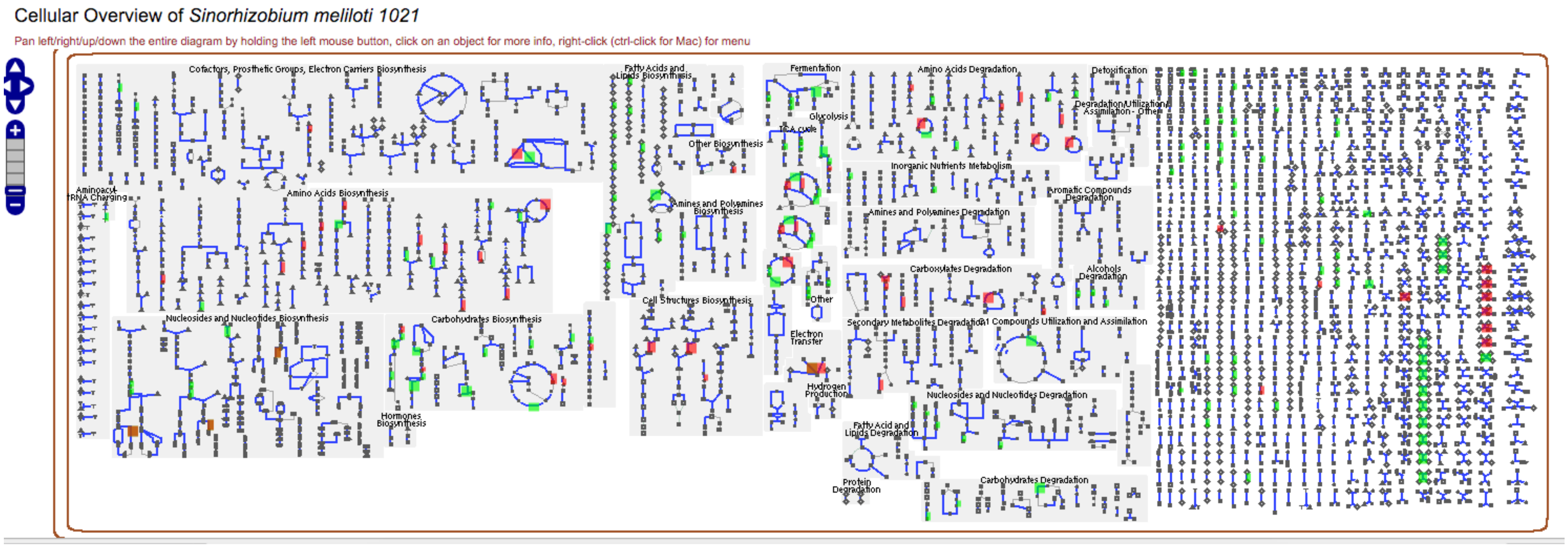

***S. americanum* CCGM7.**

Spot no.	ID BIOCYC	Spot no.	ID BIOCYC
2	SMc03846	21	SMc02755
3	SMc02483	14	SMc00574
4	SMc00149	1	SMc02692
8	SM_b20025	7	SMc01350
11	SMc02562	23	SMc00324
12	SM_b20891	9	SMc00913
20	SMc02501	25	SMc02433
28	SMc01920	5	SMc00090
29	SMc02689	13	SM_b21432
31	SMc02499	22	SMc01657
16	SMc01215	27	SM_b21432
17	SMc02717	34	SMc01657
18	-	6	SMc04454
26	SMc01770	10	SMa1303
19	SMc04018	15	-
35	SMc04088		
24	SMc03925		
30	SM_b20895		
32	SMc03983		
33	SMc03979		

***S. americanum* CFNEI73.**

Spot no.	ID BIOCYC	Spot no.	ID BIOCYC
37	SMc02487	58	SM_b20615
44	SMa1927	64	SMc02599
47	SMc00869	48	SM_b21181
53	SMc02377	46	SMc01312
54	SMc02581	42	SMc00100
57	SMc00781	39	-
70	SMc01915	50	SMc01027
72	SMc02465	67	SMc01861
36	SMc03131	40	SMc02083
43	SMc01801	61	SMc01905
49	SM_b21163	68	SMc02146
51	SMc04455	45	SMc02978
52	SMc02580	55	SMc04383
59	SMc03112	38	SMc00168
63	SMc01732	60	SMc01043
65	SMc04325	56	SMa1315
69	SMc02832	41	SM_b20964
71	SMc00951		
62	SMc03981		
66	SMc03926		

Supplementary Fig. 3. Metabolic reconstruction with BioCyc using the most abundant proteins of the strains detected in the proteome. A, *Rhizobium phaseoli* strains. Enzymes participating in metabolic pathways. Green boxes, the most abundant proteins from CCGM1 strain. Red boxes, the most abundant proteins from CIAT652 strain. B, *Sinorhizobium americanum* strains. Enzymes participating in metabolic pathways. Green boxes, the most abundant proteins from CCGM7 strain. Red boxes, the most abundant proteins from CFNEI73 strain. Brown were both coincided. The lists of proteins enable their use directly on the BioCyc page with the denoted organism. (-) denotes that no homolog was found in the strain.
